# Investigating Extracellular Vesicles in Viscous Formulations: Interplay of Nanoparticle Tracking and Nanorheology via Interferometric Light Microscopy

**DOI:** 10.1002/smsc.202400319

**Published:** 2024-11-18

**Authors:** Lucile Alexandre, Anastasiia Dubrova, Aruna Kunduru, Estelle Surply, Christopher Ribes, Imane Boucenna, Florence Gazeau, Amanda K. A. Silva, Stéphanie Mangenot, Kelly Aubertin

**Affiliations:** ^1^ Laboratoire Matière et Systèmes Complexes (MSC) Université Paris Cité, CNRS UMR 7057 45 rue des Saints‐Pères 75006 Paris France

**Keywords:** diffusion, extracellular vesicles, formulation, in situ characterizations, rheology, viscosity

## Abstract

While extracellular vesicles (EVs) demonstrate growing potential as innovative cell‐derived nanobiotherapies in diverse medical contexts, their physical properties (size, integrity, transport, etc.) in drug product formulation remain a critical concern poorly addressed so far. Herein, a methodology that relies on nanoparticle tracking analysis by interferometric light microscopy (ILM) for analyzing the concentration and size distribution of nanoparticles as well as their interactions with their local environment through a nanorheological approach is introduced. The analysis of interference patterns enables nanoparticles tracking not only in aqueous solutions but also in complex media with high‐viscosity or non‐Newtonian behavior, particularly pertinent for characterizing EV formulations. A proof of concept for in situ tracking of EVs suspended in Poloxamer‐407 as drug delivery system is presented. The ILM‐based analysis enables to 1) measure the viscosity at the nanoscale for Newtonian and non‐Newtonian fluids via calibration beads; 2) analyze data to determine the size distribution of EVs in non‐Newtonian complex fluid such as poloxamer formulation, and 3) analyze the interactions of EVs with poloxamer‐407. The proposed approach represents a valuable tool to understand the nanorheological behavior of EVs in viscoelastic media in situ as well as a quality control test for EV formulations intended to clinical use.

## Graphical abstract

Nanoparticle tracking analysis by interferometric light microscopy is a methodology for analyzing the concentration and size distribution of nanoparticles as well as their interactions with their local environment through a nanorheological approach. The analysis of interference patterns enables nanoparticles tracking not only in aqueous solutions but also in complex, high‐viscosity, or non‐Newtonian media, particularly pertinent for characterizing extracellular vesicle formulations.

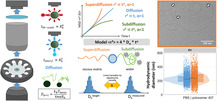

An introduction to the terminologies used in this manuscript, including definitions of nanorheology, MSD, and viscosity, is provided in the Table S1, Supporting Information.

## 1 Introduction

Extracellular vesicles (EVs) are membrane‐bound vesicles ranging from tens to hundreds of nanometers released by any types of cells in biofluids and tissues. Encapsulating proteins, lipids, and nucleic acids^[^ ^1^ ^]^ sorted from the secreting cells, EVs play a crucial role in intercellular communication. They travel easily across tissues with the potential to target distant cells, delivering their cargo specifically.^[^ ^2^ ^]^ The transported molecules reflect the biological signature and environmental cues of the cell from which they originate. It has been shown that EVs participate in many, if not all, physiopathological processes, such as the embryonic development,^[^ ^3^ ^]^ the progression and resistance of cancers^[^ ^4^, ^5^ ^]^ or the initiation of a prometastatic niches distant to primary tumor.^[^ ^6^ ^]^ To achieve their unique role of nanovectors for intercellular communication, EVs must cross many different complex media (extracellular matrix, blood, and other biofluids…) where they are engaged in multicomponent interactions. Proteins can bind to EVs forming the so‐called protein corona that will influence their transport properties and their capabilities to be internalized by recipient cells. EVs also travel across tissues, where pores can be smaller (down to 20 nm) than the average size of EVs (≈20–1000 nm).^[^ ^7^, ^8^, ^9^ ^]^ This transport relies on the respective physical and biological properties of the extracellular matrix and of the EVs and is driven by the intimate molecular interactions between them. These characteristics, along with their intrinsic biological activity, make EVs promising candidates as biomarkers^[^ ^10^ ^]^ and as endogenous drug delivery nanosystems. However, the interplay of EVs with the surrounding complex biological medium, the related transport properties of EVs, and the dynamics governing this transport are not well explored.^[^ ^8^, ^11^ ^]^

Considering a therapeutic setup, the formulation of EVs to constitute the drug product may play a decisive role in the therapeutic outcome. The formulation choice is primarily driven by preservation requirements and the administration strategy. Several studies have focused on EVs formulated in dispersions based on Poloxamer 407,^[^ ^12^ ^]^ chitosan,^[^ ^13^ ^]^ silk fibroin,^[^ ^14^ ^]^ etc.

Notably, our team has focused on the local application of EVs derived from mesenchymal stem cells within a thermoactuated Poloxamer 407 for fistula healing. The thermoresponsive formulation enabled the administration of EVs at the liquid state (<20 °C) in the site of interest while the liquid–gel transition (see phase diagram of Poloxamer 407 in Figure S1, Supporting Information) at body temperature enabled the sustained release of EVs locally^[^ ^14^ ^]^, thereby contributing to the healing process.^[^ ^15^, ^16^ ^]^ The formulation itself favored fistula healing probably via a mechanical occlusive effect.^[^ ^16^ ^]^ Despite these interesting results, little is known about the interplay of EVs and the viscous matrix, their transport, and interactions within the fistula environment characterized by constant fluid secretion from the digestive tract.

Despite the wide range of applications of EVs as biotherapies and drug delivery vectors, the characterization of EVs in complex media such as the extracellular matrix or a pharmaceutical formulation remains challenging because of the difficulty to monitor EVs at the relevant nanoscale resolution and in a dynamic way. The nanometric scale of EVs, falling below the limit of diffraction, precludes standard optical microscopic imaging technique. Different technologies have been investigated to face this challenge. Transmission electron microscopy (TEM) and cryo‐electron microscopy (CryoEM) are powerful techniques for the characterization of EVs at the nanoscale.^[^ ^17^ ^]^ However, TEM has to be performed on dehydrated sample, which precludes any in situ size and morphology measurement. With this limitation, CryoEM appears as a good alternative, as it preserves EVs in a near‐native state by rapidly vitrifying the sample, providing detailed insights, and is particularly valuable for studying EVs within complex media. However, CryoEM is a costly technique associated with low accessibility and therefore limited utility as a quality control test for formulations intended to clinical use. In contrast, the nanoparticle tracking analysis (NTA) methodology, relying on particle detection and Brownian motion analysis in a purely viscous medium, enables real‐time quantification of particle concentration and determination of hydrodynamic radius following particle tracking within a small observation chamber. The nanoflow cytometry gives access to the same information but relies on rapidly flowing fluid stream measurement and is based on the intensity of forward scattered signal after calibration with beads of known size. However, due to the presence of millimetric capillaries, these technologies are limited to characterizing EVs within a very low viscous suspension (typically viscosity of water). There is therefore a critical need for advanced and straightforward methodological approaches to study the interactions of EVs with a complex environment.

**Figure 1:**
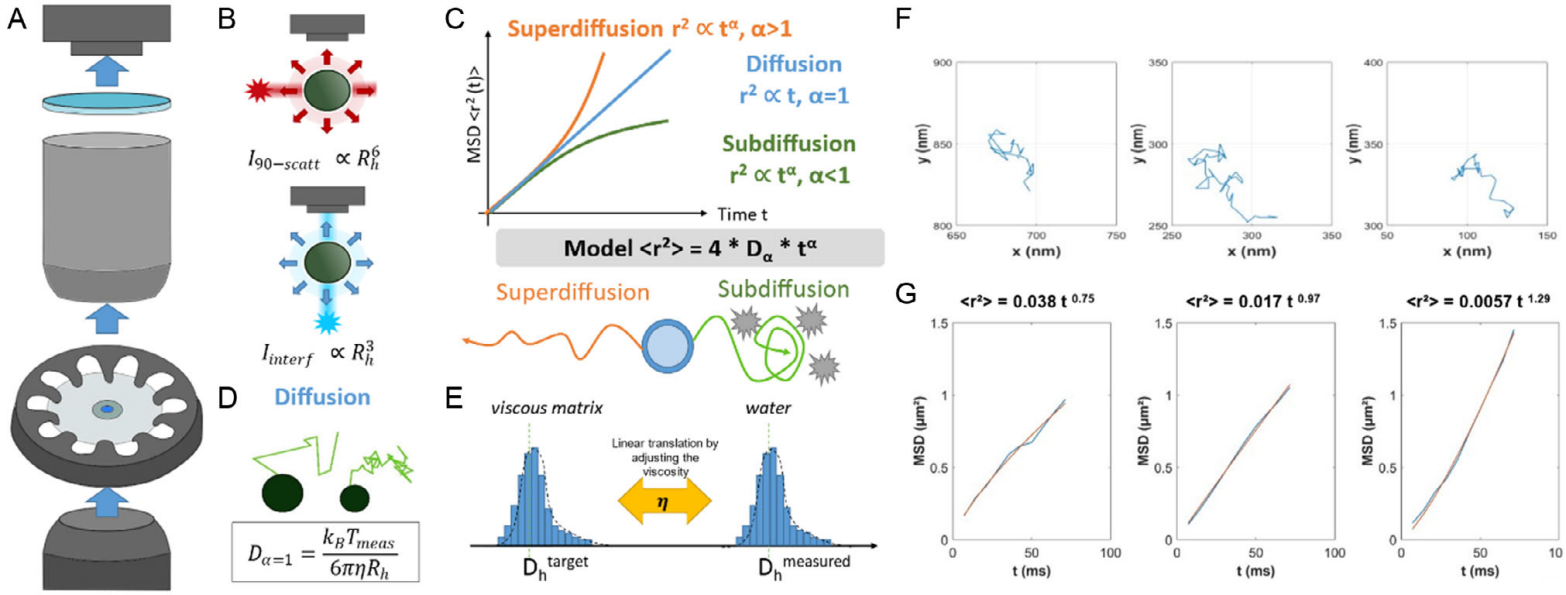
Presentation of the ILM technique for in situ nanoparticle tracking analysis and characterization in complex media. A) A closed‐up view of the light path (blue arrows) from the light source, through the sample, to the detection. B) Schematic representation of the light path for the ILM (blue arrows) compared to the light path for the conventional NTA (red arrows), illustrating a higher light intensity and therefore a better contrast for ILM. C) In a complex viscoelastic medium or upon interactions of the particle with its surrounding medium, the motion of the particle, characterized by its mean square displacement as a function of the time, can be diffusive (Brownian motion), superdiffusive, or subdiffusive, resulting in a power law with *α* value equal to 1, larger or smaller than 1, respectively. D) In a purely viscous fluid (*α* = 1), nanoparticles undergo a Brownian motion, whose analysis gives access to the nanoparticle hydrodynamic radius ($R_{h}$) through the Stokes–Einstein equation, where $D_{\alpha =1}$ is the diffusion coefficient, *k*_*B*_ the Boltzmann constant, $T_{\text{meas}}$ the temperature (measurable parameter), *η* is the dynamic viscosity of the carrier fluid, and $R_{h}$ the hydrodynamic radius. E) In the case of a particle with a known hydrodynamic size, the measurement of the MSD can allow access to dynamic viscosity (*η*) through the Stokes–Einstein equation. Indeed, the translation of the size distribution toward the known $R_{h}$ value allows then to access the local viscosity (*η*) seen by the particle. F) Trajectories of three particles with G) their MSD (blue) and the fit of the MSD (orange), with the corresponding fitting power law.

In this article, we propose an innovative approach that allows to investigate the interactions of EVs with a complex medium. We are using interferometric light microscopy (ILM) to detect and track the nanoparticles motion at the nanoscale (**Figure** **1**A,B). ILM was initially developed to characterize the size and concentration of (bio)nanoparticles, such as viral vectors in aqueous medium.^[^ ^18^ ^]^ ILM was also used to investigate EVs in aqueous buffer^[^ ^19^ ^]^ or a diluted plasma.^[^ ^20^ ^]^ This technology has been validated by numerous references for EV characterization, but until now, its use has been limited to the quantification and size distribution of particles.^[^ ^21^, ^22^, ^23^ ^]^ ILM uniquely relies on the interference pattern of the transmitted light to detect the particle, while standard methods use the scattered light intensity in the orthogonal direction under laser illumination. The transmitted signal (proportional to $R_{h}^{3}$ for ILM) has an intensity higher than the 90°‐scattered signal (proportional to $R_{h}^{6}$) in case of standard NTA methodology. The analysis of the forces exerted by a liquid on a solid particle allows us to connect the movement of the particles and the physical properties of the fluid. As for other NTA methods, ILM relies on the Brownian motion of spherical particles to give access to their hydrodynamic size, according to the Stokes–Einstein equation (Equation (1)).

(1) $$D\;=\;\frac{k_{B}T_{\text{meas}}}{6\;\pi \;\eta \;R_{h}}$$

where *D* is the diffusion coefficient, $k_{B}$ the Boltzmann constant, $T_{\text{meas}}$ the temperature measured during the experiment, *η* the dynamic viscosity of the carrier fluid, and $R_{h}$ the hydrodynamic radius of the particle. This relationship linking the diffusion coefficient to the inverse of particle hydrodynamic size supposes a purely viscous medium in which particles undergo a free diffusive motion (Figure 1C), a supposition that was never challenged in previous publications. This relationship can then give access to 1) the hydrodynamic radius whenever the viscosity is known or 2) the viscosity of the carrier fluid whenever the particle size is known. In standard NTA experiments in an aqueous suspension of known viscosity, the number of detected particles such as EVs can be quantified and their size distribution is derived based on the tracking of each particle trajectory (Figure 1F). However, within a more complex viscoelastic matrix, the particle detection might be more challenging and the particle trajectory might deviate from a purely diffusive motion at certain temporal and spatial scales. If the particle motion is still purely diffusive within a specific time window and in a restricted volume, the particle probes the local viscosity of the surrounding environment. In other situations of particle interactions with the matrix, the motion can be subdiffusive (Figure 1C), indicating that the Brownian motion is hindered by obstacles or mechanical traps. Interactions at the nanoscale can also promote intermittent ballistic motion of the particles, which gives rise to superdiffusion (Figure 1C). All these local regimes of motion can be evidenced by measuring the mean square displacement (MSD) of each particle (Figure 1G, Equation 2). The MSD is a statistical metric used to quantify the average squared displacement of a particle from its initial position over time. The temporal dependence of the mean of the MSDs can be approximated by a power law as a function of the time (Equation 3) with an anomalous diffusion exponent *α* = 1 for a Brownian diffusion motion (Stokes‐Einstein Equation 1), *α* < 1 for subdiffusive motion and *α* > 1 for superdiffusive motion

(2) $$<\Delta r^{2}\left(t\right)>=<{\left[x\left(t+t\prime \right)-x\left(t\prime \right)\right]}^{2}+{\left[y\left(t+t\prime \right)-y\left(t\prime \right)\right]}^{2}{>}_{t^{\prime }}\;$$

(3) $$\text{MSD}\left(t\right)=<\Delta r^{2}\left(t\right)>\;=4D_{\alpha }\;t^{\alpha }$$

where $r^{2}$ is the space explored by the particle during an interval of time *t* (in *s*), *α* is the anomalous diffusion exponent, and $D_{\alpha }$ is the generalized diffusion coefficient (in ${\text{μm}}^{2}.s^{-1}$ for diffusive behavior and in ${\text{μm}}^{2}.s^{-\alpha }$ otherwise) (Figure 1D). In the case of Brownian motion, $D_{\alpha =1}=D$ is defined in Equation (1). Equation (2) describes the MSD of one particle in two dimensions with an average along the trajectory. MSD characterizations provide invaluable information on the interactions between the nanoparticles and their environment,^[^ ^24^, ^25^, ^26^ ^]^ in an approach coined nanorheology, as it was described in the literature for intracellular inert or biological nanoprobes.^[^ ^27^, ^28^ ^]^ In the context of ILM experiment, the measurement of the diffusion coefficient is then related to the size of the particles as described by Equation (1).

To our knowledge, nanorheological approaches focusing on the diffusion of EVs in a complex medium have never been developed in the field of EVs due to technical challenges. Contrary to other NTA systems relying on detection inside a microfluidic chamber in which the sample is injected through milli‐ or microchannels, ILM detection is performed by simple deposition of the sample on a glass slide. The system of ILM is thus able to work not only in aqueous solutions but also in a complex medium with relatively high viscosity or with viscoelastic behavior. Here we take advantage of such ILM technology to investigate the transport properties of EVs in poloxamer 407 formulation and to probe their interactions with this pharmaceutical carrier medium in comparison to aqueous suspension. We first developed an ILM‐based methodology using synthetic beads of various known sizes to measure the nanoscale local viscosity experienced by the beads in different media (Figure 1E). After this rheological step, we ground on this strategy to perform in situ nanorheological analysis by tracking EVs in complex viscous media in order to understand the impact of a hydrogel formulation on EV transport properties as well as size and concentration.

## 2 Experimental Section

### 2.1 Chemicals

Phosphate‐buffered saline (PBS), glycerol (reference 8.18709, CAS number 56‐81‐5), and Poloxamer 407, named also pluronic F‐127 (reference P2443, CAS number 9003‐11‐6), were obtained from Sigma‐Aldrich (St‐Louis, MO, USA).

Glycerol was diluted in milliQ Water to reach different concentrations of 0.94, 1.88, 3.75, 7.5, 15, 30, 40% w/v for macroscopic rheological measurements. For ILM measurements, glycerol solutions were prepared at 2X concentrations (1.88–80%) with serial dilutions and mixed with 2X concentrations of polystyrene (PS) beads or EV suspension, to reach final concentrations of glycerol between 0.94% and 40%.

Poloxamer 407 was resuspended in PBS 1X at stock concentration of 20% w/v and stirred at 4 °C for 24 h. The poloxamer solution was then filtered with 0.22 μm filter for sterilization. The stock solution was then serial diluted to reach concentrations of 0.12, 0.23, 0.47, 0.94, 1.88, 3.75, 7.5% for macroscopic rheological measurements. For ILM measurements, Poloxamer 407 solutions were prepared at 2X concentrations (0.24–15%) and mixed with 2X concentrations of PS beads or EV suspension, to reach final Poloxamer 407 concentrations between 0.12 and 7.5%. The Poloxamer 407 dilutions were kept at 4 °C and mixed with PS beads or EV suspension without reheating step and extemporaneously right before ILM measurements.

Cut micropipette tips were used to sample all viscous solutions.

### 2.2 Polystyrene (PS) Beads

Beads were selected from the 3000 Series Nanosphere Size Standards from Thermofisher Scientific (Waltham, MA, USA), calibrated by the supplier within nanometer‐scale dimensions using the trackable National Institute of Standards and Technology methodology. They had a density of 1.05 g cm^−3^ and an index of refraction of 1.59 at 589 nm (25 °C). PS beads stock concentration was retrieved from information given by the supplier.

### 2.3 Cell Culture and EVs Production

EVs were produced from primary human adipose‐derived stem cells (hADSC) in turbulence^[^ ^29^ ^]^ (proprietary technology, WO2019002608). Cells were cultured in complete DMEM medium (Dulbecco's Modified Eagle Medium), that is, DMEM (Gibco, Life Technologies Corporation, U.S.A) supplemented with 10% FBS (Fetal Bovine Serum, Corning) and 1% of penicillin/streptomycin (Gibco) at 37 °C with 5% of CO_2_. All cells were cultivated in T150 flasks and subcultured when reaching 80% of confluency for cell expansion. Prior to EV production, cells were detached with 0.05% trypsin and transferred in a 500 mL spinner flask (Bellco, 1967‐01000) containing microcarriers. Cell seeding was done at a ratio of 10 cells for 1 microcarrier in complete DMEM. Turbulence EV production process was performed in starvation medium (no serum) after three rinsing steps with PBS. The conditioned medium was collected after 4 h of turbulence (34–36 μm Kolmogorov length) and transferred in 50 mL Falcon tube for clarification step using sequential centrifugations at room temperature: the supernatant was retrieved after a run at 300 g for 5 min and again after a run at 2000 g for 10 min (Eppendorf 5702). The EVs were then purified by tangential flow filtration (TFF) using a cellulose T‐Series cassette (Cytiva, DC030T02). The concentrated product was diafiltered using PBS 1X. The EV‐rich sample obtained after TFF was filtered with a 0.22 μm filtration units. The concentration of each postpurification EV suspension was measured by NTA (Nanosight NS300, Malver Panalytical) for quality control. Each sample was diluted in PBS1X to reach the concentration range recommended by the manufacturer (from 1 to 5 × 10^8^ particles/ml) and five videos of 60 s were acquired and analyzed. The EV‐enriched fraction was stored at −80 °C prior ILM characterization using Videodrop (Myriade).

### 2.4 ILM Measurement

ILM detection relies on the detection of the signal from nanoparticles directly on the optical path (180°) (Figure 1B), taking advantage of the interferences between the scattered and the LED source light. The intensity of the interferences between the light source and the scattering light from the particles on the optical path can be described as

(4) $$I_{\text{interf}}=\;\left|E_{\text{source}}+\;E_{\text{scatter}}\right|\;$$

(5) $$I_{\text{interf}}=\;I_{\text{source}}+\;I_{\text{scatter}}+2\sqrt{I_{\text{source}}*I_{\text{scatter}}}\;\left(\theta_{\text{source}}-\;\theta_{\text{scatter}}\;\right)$$

with $I_{\text{source}}$ and $E_{\text{source}}$ the light source intensity and field, $I_{\text{scatter}}$ and $E_{\text{scatter}}$ the intensity and the field of the scattering light from the particles, and θ the phase of each light source.^[^ ^29^ ^]^

As described in Figure 1A,B, the light source, sample, and camera detector were aligned for the detection of interference patterns. The signal strength was then proportional to the 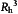, mitigating the signal weakness associated with small particles.

ILM measurements were performed using Videodrop instrument^[^ ^30^ ^]^ (Myriade, Paris). The sample chip was washed with ethanol and distilled water before and after each measurement. A volume of 7 μL was deposited on the center of the sample chip for each sample measurement. The chip was then loaded on the optical path. Given the significant impact of temperature on measurements, temperature was measured for each measurement and adjusted in the software for integration into the size distribution analysis. Recordings were performed using the QVIR software, with a minimum of 300 particles followed per video. The measurement window spanned a surface of 10.9 × 10.9 mm, with a size of the pixel on the detector of 10.6 × 10.6 μm. Measurements were taken with an optical magnification of 187.5X, which gave a measurement window of 58 × 58 μm and a pixel size of 56.7 × 56.7 nm. The time interval between successive images was 7.15 ms, which included an exposure time of 0.9 ms.

### 2.5 Data Analysis

All data analyses were performed with MATLAB (R2021a) using inbuilt functions. For each individual object, knowing its *x* and *y* positions over time, the trajectory coordinates were retrieved from the position of particles as function of time (extracted from coordinates.csv file exported using QVIR software). The MSD was then calculated using Equation (2).

The MSD analysis was conducted using the ‘fitnlm’ function of MATLAB, focusing solely on the initial ten data points due to the reduced statistical significance resulting from fewer data points available for analysis. Indeed, as trajectories consisted of a maximum of 100 data points (if the particle was tracked throughout the entire observation period) and a minimum of 10 points, diminishing statistical significance over time led to increased noise in the MSD curve at later time points. For each condition, we plotted the geometric average of the MSD over the *N* (>300) particles that were monitored. The plots were made using the function ‘shadedErrorBar’^[^ ^31^ ^]^ to display the standard error of the mean (SEM). As MSDs followed power laws (Equation (3)), for each condition and for each particle, a diffusion coefficient $D_{\alpha }$ and an anomalous diffusion exponent α were extracted. All the average values of these two parameters can be found in Table S2, Supporting Information. The averaged generalized diffusion coefficient corresponds to the geometrical mean of the generalized diffusion coefficients of all particles, and the averaged anomalous diffusion exponent corresponds to the arithmetic mean of the anomalous diffusion coefficients of all particles.

Size distributions were illustrated using the function ‘al_goodplot’.^[^ ^32^ ^]^ Raw data points are shown as colored dots. The lowest colored shape represents the kernel density of the size distribution. The second, brighter colored shape represents the standard deviation around the mean, which was indicated by a black star. The brightest shape represents the first and third quartiles around the median, depicted by a black line. This final shape was pinched around the 5% confidence interval around the median.

### 2.6 Macroscopic Viscosity Measurement

Macroscopic viscosity measurements were performed using a Physica RheoCompass MCR 302 (Anton Paar, France) equipped with a solvent trap for preventing solvent evaporation. The measurements were performed using a cone and plate geometry (diameter = 50 mm, cone angle = 1°). Temperature was controlled during measurement using a Peltier plate unit adjusted at 24 °C and was regulated during 3 min prior each measurement. Poloxamer 407 viscosities were measured using a shear gradient of 100 (1/s) during 3 min at concentrations of 0.94, 1.87, 3.75, 7.5, 20, and 30%. Glycerol viscosities were retrieved from the literature.^[^ ^33^ ^]^

### 2.7 Cryo‐Electron Microscopy Imaging

Grids for cryoEM were prepared using an automated plunge freezer (EM‐GP, Leica). 3.5 μL of samples were deposited on glow‐discharged lacey holey carbon grids (Ted Pella INC., Lacey grids) equilibrated at 10 °C. Samples were blotted during 3–5 s to remove the excess sample and leave a thin film in the carbon hole. The blotting was carried out on the opposite side from the liquid drop and plunge‐frozen in liquid ethane at −181 °C. For high‐viscosity samples, an equilibration time of 10–15 s was added and blotting time was adapted to the viscosity of the sample, from 3 to 20 s. The samples were observed at liquid nitrogen temperature using a Tecnai F20 (Thermofischer, FEI, Eindhoven, the Netherlands) microscope operated at 200 kV and equipped with a Falcon II direct electron detector (Thermofischer, FEI, Eindhoven, the Netherlands). The data were collected at a magnification of 50 000 resulting in a size of 2 Å per pixel.

## 3 Results

### 3.1 Evaluation and Calibration of the ILM NTA Technique on 80–400 nm Polystyrene Beads in Aqueous Suspension

**Figure 2:**
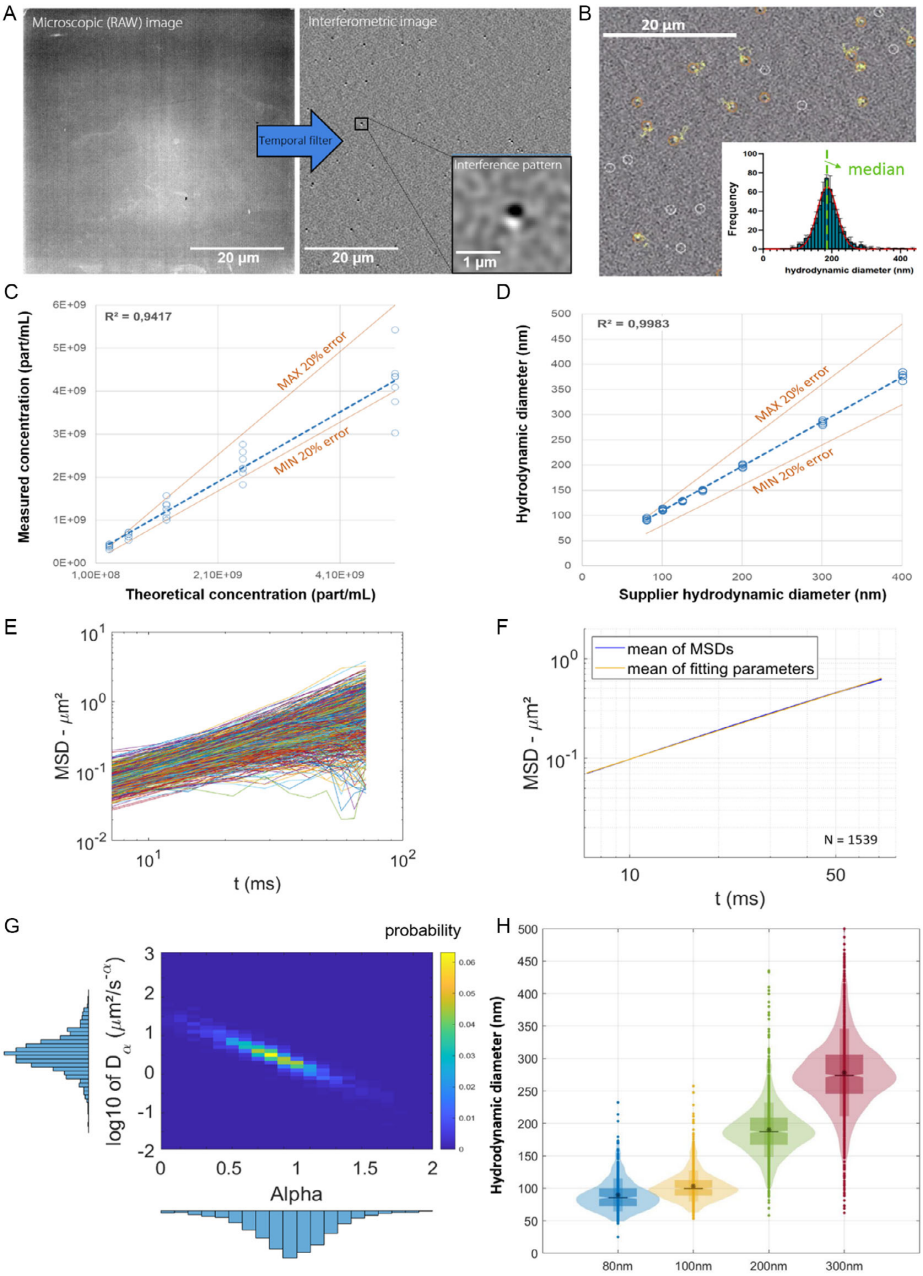
Description and characterization of the ILM instrument for PS beads quantification and sizing in water. A) Interferometric image generated from the wide field (microscopic image) using temporal filtering and allowing to visualize interferometric pattern related to nanoparticles. B) Typical ILM images with particle tracking of each individual particle plotted in yellow. Inset: Median size distribution determined by fitting of Equation (1). The measured C) concentration and D) size calculated for beads with the ILM instrument are in perfect agreement with theoretical values. E) Representation of the MSDs of a sample of 200 nm beads in water with the corresponding (1539 MSDs are plotted on the graph). F) Mean MSDs of 200 nm beads in water (in blue) with the SEM distribution around it and the equation made of the mean of each parameter (in orange) (*N* = 1539 beads). G) Distribution of diffusion coefficients ($D_{\alpha }$) and the anomalous diffusion exponent (*α*) extracted from each measured particle of the sample plotted for 1539 beads. H) Representation of size distributions of the 80, 100, 200, and 300 nm‐diameter beads in water (for *N* = 509, 1028, 1539, and 1536 respectively).

To ascertain the ILM methodology, NTA measurements were first conducted on a solution of monodisperse PS beads of 80–400 nm nominal diameter in water. In this experiment, the hydrodynamic radius of the particles ($R_{h}$) and the viscosity (*η*) of the carrier fluid, two parameters of the Stokes–Einstein equation (Equation (1)), are known and used to validate our scheme of analysis in a purely diffusive medium. These beads serve as exemplary standards with known geometrical diameters given by the manufacturer (i.e., 80 ± 3 nm, 100 ± 3 nm, 203 ± 5 nm, 303 ± 6 nm, and 401 ± 6 nm for the 80, 100, 200, 300, and 400 nm bead, respectively). Dilution in water was performed for each particle size to achieve the instrument's measurement range for assessment of the size distribution (**Figure** **2**C).

The ILM technique enables the detection of particles as presented in Figure 2A,B. Custom image processing, developed by Myriade,^[^ ^30^ ^]^ is employed on each image to isolate and subtract the static signal emanating from the LED, from the dynamic signal generated by spontaneous particle movements due to their Brownian motion (Figure 2A). A distinct pattern made of a white and black doublet represents interference patterns between the source and the scatter signal produced by the particle (Figure 2A), whose position can then be precisely extracted over time. Particles must stay in focus long enough (at least ten successive images) to be able to be tracked by the system and thus analyzed for MSD measurement. If it is not the case, particles are not considered for diffusion measurement and size quantification. These two kinds of particles are identified with orange and white circles respectively on Figure 2B.

By conducting measurement within a known volume of the sample, this method provides direct access to the concentration (in part/mL). Moreover, if working with a sample of known or precalibrated viscosity, the particle size distribution can be inferred from the Brownian motion recorded for each particle and there is use of the Stokes–Einstein equation (Equation (1)), also presented on Figure 1C.

We first checked the linearity of ILM measurements to detect particle concentration across a range of nominal concentration from 10^8^ to 10^10^ part/mL (Figure 2C). Besides, in this range of concentrations, the hydrodynamic size was deduced from the recording of each particle Brownian motion and application of Stokes–Einstein Equation (1) considering a viscosity equal to 1 mPa.s for water. As shown in Figure 2D, the obtained size distributions were fully consistent with nominal diameters indicated by the supplier in the range of 80–400 nm. Notably, accuracy remained within a 10% error range for sizes ranging from 200 to 400 nm and within 20% for 100 nm beads. Both concentration and size analyses demonstrated strong linearity, with high coefficients of determination (*R* ^2^ = 0.942 for concentration and *R* ^2^ = 0.998 for size). The system showed good accuracies, with deviations of ±10% for size and ±20% for concentration. On average, the coefficient of variation is below 5% for size measurements and below 20% for concentration measurements, demonstrating good repeatability. Further analyses were performed with beads of 100, 200, and 300 nm in diameter, mimicking the size of EV samples of interest.

For the Stokes–Einstein equation to be applicable, the particles should undergo purely diffusive motion in a uniform viscous medium. To ensure that these conditions were met, the MSDs of each particle were measured, as depicted in Figure 2E,F. Our experimental window was reduced to the first 10 points of the MSD for each particle to reduce edge effects.^[^ ^34^ ^]^ Regarding data collection size, each MSD result was obtained for at least 500 particles for each condition. The mean of the MSDs is depicted in blue on Figure 2F, along with the SEM around it. In yellow, the power law corresponding to the geometric mean of diffusion coefficients ($D_{\alpha }=2.70+0.11/-0.04{\text{ μm}}^{2}s^{-1}$) and arithmetic mean of anomalous exponents (α=0.95±0.01) is represented, showing a very good adjustment to the experimental curve. The distribution of diffusion coefficients versus anomalous coefficient of each particle is illustrated in Figure 2G and Figure S2, Supporting Information. A correlation between the diffusion coefficients and anomalous diffusion exponents was observed for particles of all three sizes. Indeed, we observed a decrease in $D_{\alpha }$ while *α* decreases. This correlation could be caused by a variability of the behaviors around the diffusive one (α=1), as presented on Figure S3, Supporting Information. Indeed, when looking individual MSDs of particles on log–log scale, we observe similar general behavior with a common crossing point for all MSD (around *t* = 20 ms for this condition). This means that the majority of the MSD with *α* values slightly above 1 have a low $D_{\alpha }$ and inversely.

In each condition and for each size of beads, the anomalous diffusion exponent was close to 1 demonstrating movement very close to purely diffusion motion and confirming the validity of Stokes–Einstein equation for displaying the size distributions of 80, 100, 200, and 300 nm (Figure 2H).

**Figure 3:**
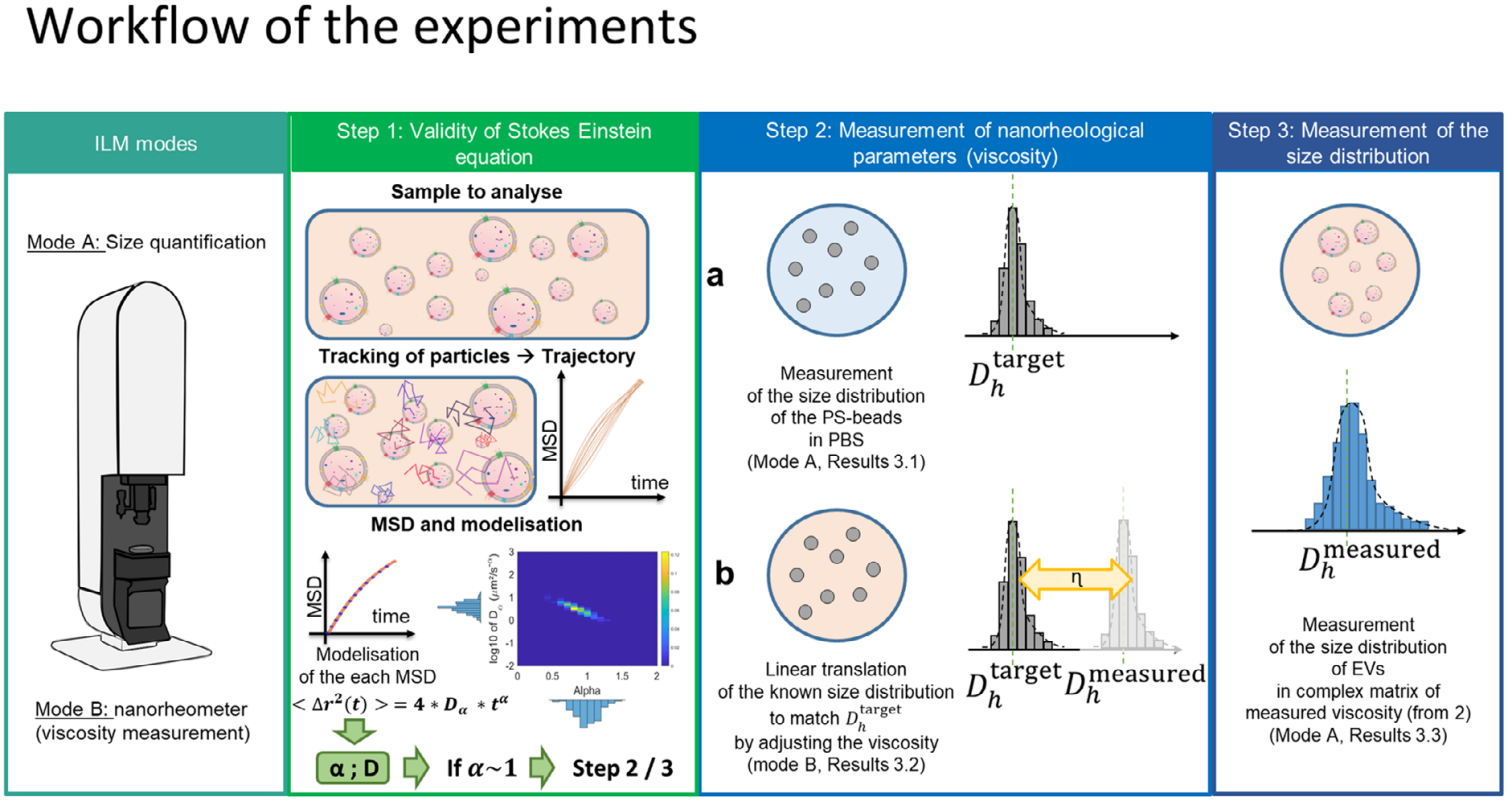
Description of the experimental workflow. The ILM instrument can be used for either size quantification or as a nanorheometer following two or three steps. Step 1: To analyze a sample of particles in a complex matrix, we first analyzed their individual trajectories to obtain the corresponding MSDs. By modeling each of these MSDs by a power law, we obtained the $D_{\alpha }$ and *α* coefficients, represented by heatmaps, as explained in Section 3.1. For *α* coefficients close to 1, a condition that was confirmed in all experiments of this study, the Stokes–Einstein equation links the hydrodynamic size of each particle to the viscosity of the medium. Step 2: ILM instrument can be used to measure nanorheological properties (viscosity) of an unknown matrix. (a) We first determined the size distribution of probe particles (calibrated‐size beads) in PBS. (b) Then the probe particle trajectories are analyzed in a matrix of unknown viscosity checking the validity of Stokes–Einstein equation (Step 1). Then the viscosity is adjusted in the equation so that the size distribution in this matrix of unknown viscosity matches the probe size distribution in PBS. This allows to determine the local viscosity of the matrix probed by nanoparticles of defined size as described in Section 3.2. Step 3: The ILM method can be used to determine the size distribution of unknown particles such as EVs, whose behavior can change depending on the matrix. We again checked the MSD and validity of Stokes–Einstein equation for the particles in the matrix (Step 1) and then used the local viscosity of the matrix measured with probe beads of relevant sizes (Step 2) to deduce the size distribution of the EVs in this matrix. If the EV size distribution is different from that measured in PBS, it gives information on the interactions between EVs and the matrix as presented in Section 3.3.

Based on the characterization of the instrument and under the conditions where the Stokes–Einstein equation is valid (**Figure** **3**, step 1), ILM‐NTA can be used to determine either the size distribution or the nanorheological properties of a matrix, as shown in Figure 3. In this study, we will begin with a detailed examination of the ILM‐NTA instrument to develop a pipeline for measuring the nanoviscosity of both Newtonian and non‐Newtonian fluids using calibrated beads (Figure 3, step 2). Next, we will use the measured local viscosity to determine the size distribution of EVs within a complex matrix (Figure 3, step 3).

### 3.2 Matrix Viscosity Measurement Using Calibrated Particles (Measurement of *ɳ* When *R*_h_ Is Known)

In the case of a previously characterized diffusive motion of particles at a constant temperature, the size measurement depends solely on the dynamic viscosity of the liquid phase surrounding the particles. This nanoscopic viscosity represents the local viscosity experienced by the measured particles. While the VideoDrop instrument is typically used to determine hydrodynamic size given a known viscosity as done in Section 3.1 for the calibration beads in water, it can also be used to determine the local viscosity given a known hydrodynamic size of the particles. Indeed, when measuring the size distribution of particles of calibrated size in a viscous liquid of unknown viscosity, a linear shift in the size distribution occurs. This shift can be compensated when accounting for the viscosity of the liquid phase. The viscosity value is thus adjusted in order to match the median hydrodynamic diameter of calibrated nanoparticles to the one measured with ILM in water (Figure 1E). This step relies on a precise measurement of the size distribution of calibrated particles as done in Section 3.1.

In this part, we propose to probe the local viscosity of two different matrices: glycerol, a Newtonian fluid, and poloxamer 407, a non‐Newtonian matrix. For this purpose, the nanoprobes are the PS beads of known diameters (100, 200, and 300 nm), whose size distributions were previously determined in water (Figure 2H). We conducted ILM measurements using the PS beads in glycerol at different concentrations 0.94, 1.88, 3.75, 7.5, 15, 30, 40%.

**Figure 4:**
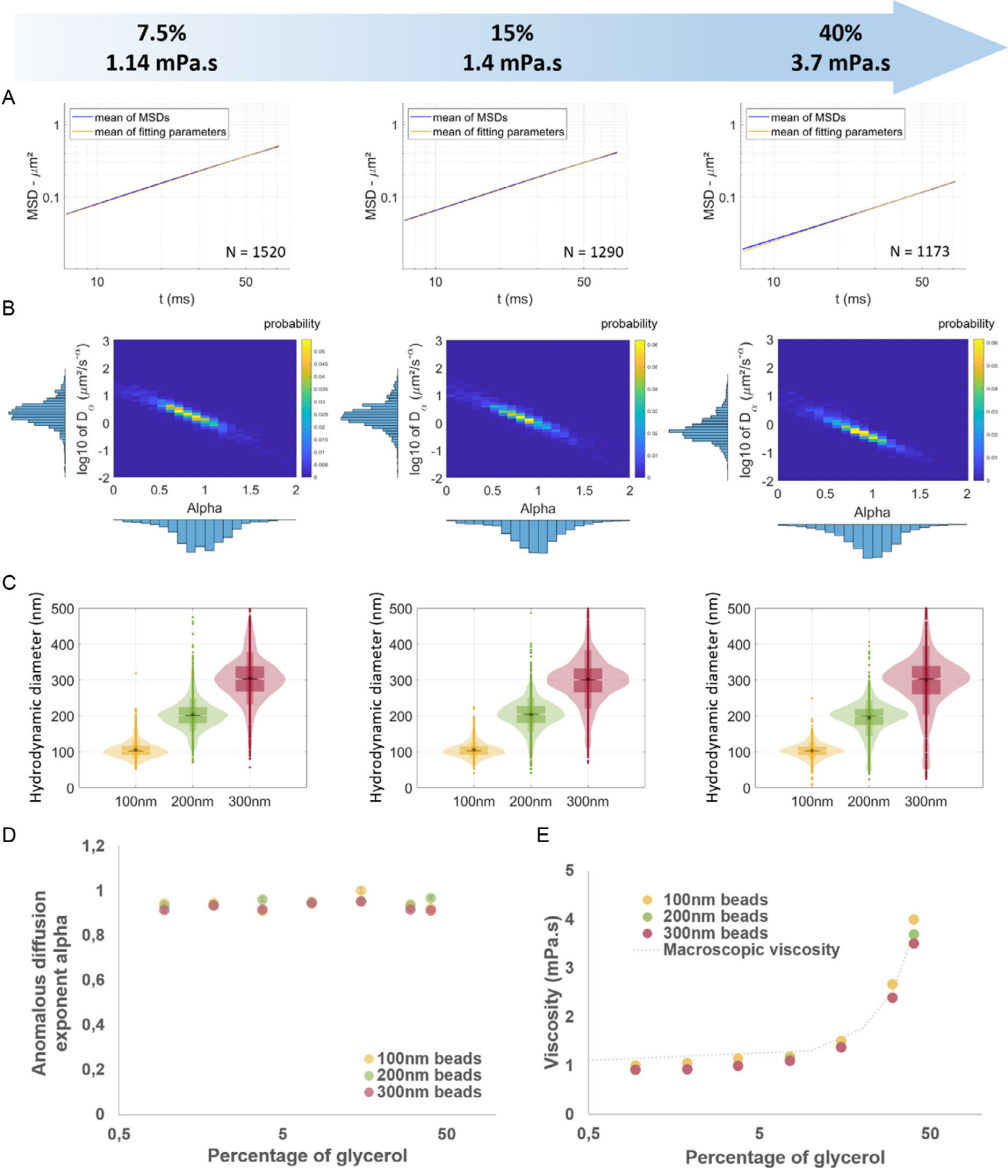
Validation of ILM as a nanorheometer in a Newtonian fluid, the glycerol. A) Representation of the mean of the fitted MSDs (±SEM) of each 200 nm particles in 7.5, 15, and 40% glycerol in blue and of the power law using the mean of the fitting parameter for each MSD in yellow. B) Representation of the distribution of the diffusion coefficient $D_{\alpha }$ as a function of the anomalous exponent α for the 200 nm‐diameter beads in 7.5, 15, and 40% glycerol. C) Representation of the size distributions deduced by ILM of 100, 200, and 300 nm‐diameter beads in glycerol at 7.5, 15, and 40%. D) Mean anomalous diffusion exponents for 100, 200, and 300 nm‐diameter beads in glycerol at different concentrations (±SEM). E) Mean local viscosity probed by the beads of 100, 200, and 300 nm‐diameter beads (*N* > 680, 1170, and 990 respectively) and measured by ILM in different concentrations of glycerol and comparison to the macroscopic viscosity measured with a clone‐plate rheometer.

To ensure the diffusive nature of particle trajectories within the glycerol matrix, we computed the MSD of each particle and fitted them with a power law, as described above (**Figure** **4**A and Figure S4, Supporting Information). Importantly, for all glycerol concentration, no difference was observed between the mean of the MSDs depicted in blue in Figure 4A for 200 nm‐diameter beads and the power law in yellow with parameters corresponding to the mean of diffusion coefficients and the mean of anomalous diffusion exponents, indicating a good concordance between the fit of the mean and the mean of the fits. The heatmap of the diffusion coefficients as a function of the anomalous diffusion exponents, displayed in Figure 4B, revealed a correlation between the two coefficients, similar to what was observed in water. As in water, the anomalous diffusion exponents *α* were close to 1 with an arithmetic mean α=0.95±0.01 in 7.5% glycerol, α=0.96±0.01 in 15% glycerol and α=0.97±0.01 in 40% glycerol, for 200 nm‐diameter beads, meaning that particles exhibit diffusive‐like behaviors by freely diffusing in the viscous fluids. As expected, the diffusion coefficients of the 200 nm diameter beads decreased from 2.20 + 0.24/−0.09 μm^2^ s^−1^ in 7.5% glycerol to 0.66 + 0.01/−0.03  μm^2^ s^−1^ in 40% glycerol, values that were consistent with the viscosity increase (from 1.14 × 10^−3^ to 3.72 × 10^−3^ Pa.s) and the literature.^[^ ^35^, ^36^ ^]^ Additionally, as expected, the diffusion coefficient decreased with increasing bead size, as shown in Figure S5, Supporting Information. A larger deviation from purely diffusion motion (Figure 4D) with an increased SEM of the MSD (Figure S5, Supporting Information) was also observed for the largest beads at the highest concentration of glycerol.

In each condition and for each size of beads, the anomalous diffusion exponent was close to 1 demonstrating movement very close to purely diffusion motion (Figure 4B,D) and confirming the validity of Stokes–Einstein equation. This allowed us to extract from the MSDs a size distribution for each particle and each glycerol concentration (Figure 4C). The bead size distribution in glycerol appears to be more spread than in water, particularly for the largest beads at the highest concentrations of glycerol. This could be explained by a decreased precision in the localization of the largest beads at the highest viscosity, which explores a smaller space between two temporal points.

The nanoscopic viscosity was obtained by matching the obtained median hydrodynamic diameter of the beads to the one measured with ILM in water. As seen in Figure 4E, the local viscosities were very similar to the macroscopic viscosity determined by a cone‐plate rheometer across the entire range of glycerol concentrations up to 40%. Discrepancies between local and global viscosity only emerged at the highest glycerol concentrations, with local viscosity slightly increasing as particle size decreased from 300 to 100 nm. This demonstrates the usability of ILM methodology to characterize the local viscosity experienced by nano‐objects.

Next, we used the same methodology and beads to investigate the nanorheological behavior, specifically the local viscosity, of a more complex and biologically relevant material, exhibiting non‐Newtonian properties, the poloxamer 407. Since the viscosity of non‐Newtonian fluids depends on temperature, shear rate, and time, we maintained a constant temperature throughout the experiments, verified by temperature measurements during each test. Each video was recorded using a freshly prepared sample to ensure consistent conditions and minimize time‐dependent effects.

Poloxamer 407 is a thermosensitive polymer that can undergo a liquid–gel transition. As the temperature increases, it transitions from a monomeric organization to micelle formation (with a characteristic size of 20 nm), eventually forming a gel. This reorganization is accompanied by a significant increase in viscosity. Here, we worked with different concentrations of poloxamer 407 (0.12, 0.23, 0.47, 0.94, 1.88, 3.75, and 7.5%) at temperatures between 20 and 25 °C corresponding to different organizations of the matrix (from monomers to micelles, see Figure S1, Supporting Information) but always in liquid phase of various viscosities.

**Figure 5:**
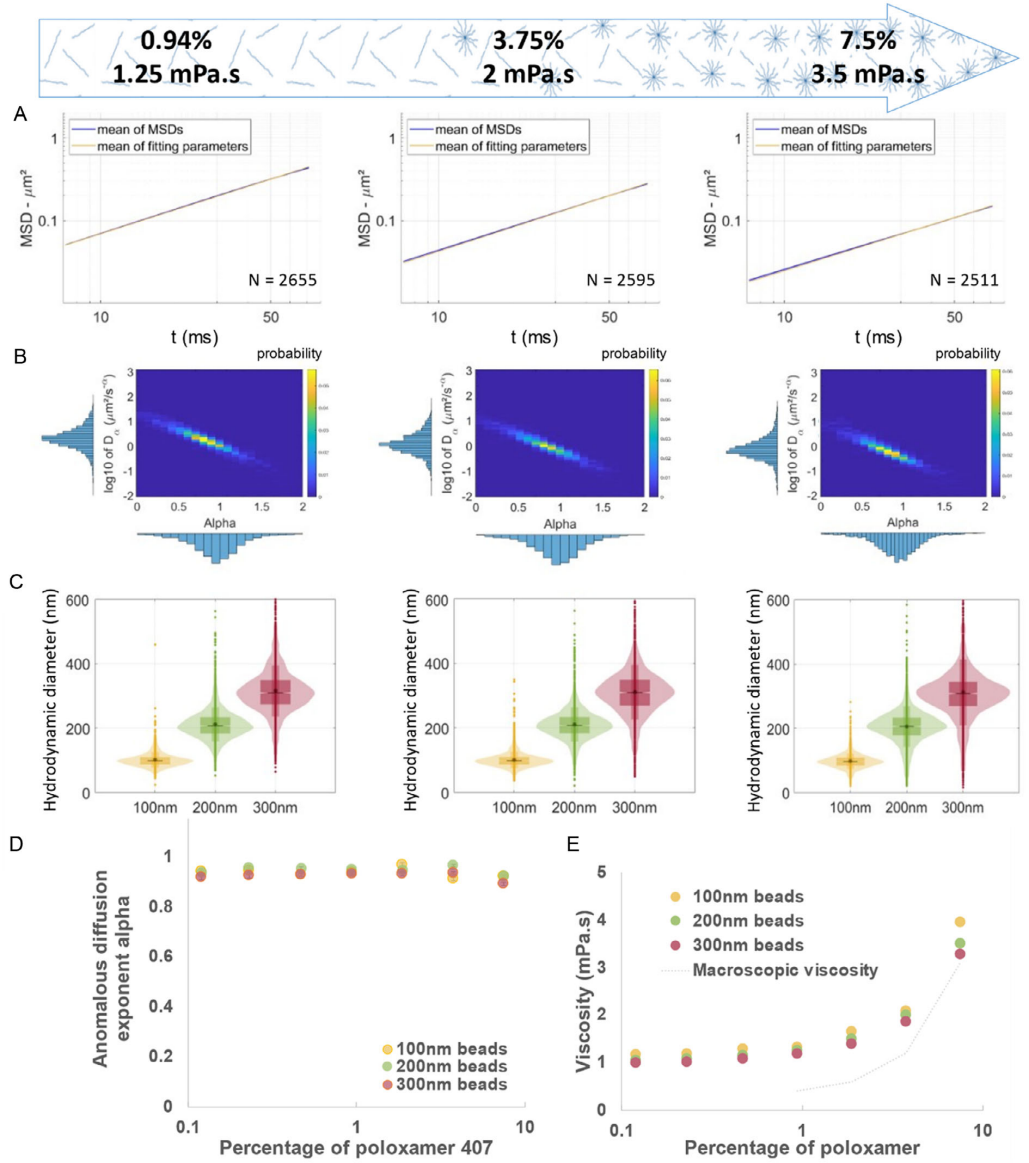
Measurement in a non‐Newtonian fluid, the poloxamer 407. A) Representation of the mean of the fitted MSDs (±SEM) of each 200 nm particles in 0.94, 3.75, and 7.5% poloxamer 407 in blue and of the power law using the mean of the fitting parameter for each MSD in yellow. B) Representation of the distribution of the diffusion coefficient $D_{\alpha }$ as a function of the anomalous exponent *α* for the 200 nm‐diameter beads in 0.94, 3.75, and 7.5% poloxamer 407. C) Representation of the size distributions deduced by ILM of 100, 200, and 300 nm‐diameter beads in poloxamer 407 at 0.94, 3.75, and 7.5%. D) Mean anomalous diffusion exponents for 100, 200, and 300 nm‐diameter beads in poloxamer 407 at different concentrations (±SEM). E) Mean local viscosity probed by the beads of 100, 200, and 300 nm‐diameter beads (*N* > 1090, 1450, and 1420 respectively) and measured by ILM in different concentrations of poloxamer 407 and comparison to the macroscopic viscosity measured with a clone‐plate rheometer.

The mean MSDs remained congruent with the power law derived from the means of each power law parameters, with $D_{\alpha }=0.74+0.02/-0.01{\text{ μm}}^{2}s^{-1}$ and α=0.92±0.01 for 200 nm diameter beads in 7.5% poloxamer (**Figure** **5**A and Figure S6, Supporting Information), validating our fitting method in a non‐Newtonian matrix. The diffusion coefficient exhibited a decrease as bead size or poloxamer concentration increased (Figure 5B and Figure S7, Supporting Information). The anomalous diffusion exponent *α* remained centered around 1 for the different concentrations of poloxamer 407 (Figure 4D and 5B), indicative of a diffusive pattern of PS beads in these poloxamer matrices.

This observation allows the extraction of a size distribution and local viscosity according to the Stokes–Einstein equation (Figures 4C and 5E). A broadening and deformation of the bead size distribution was observed at the highest concentrations of poloxamer. This phenomenon was also observed with increasing concentration (and therefore viscosity) of glycerol (Figure 5A), but to a lesser extent than in poloxamer and affects mostly the largest beads of 300 nm.

Interestingly, at similar viscosity and PS bead size constant, we observed that glycerol and poloxamer 407 exhibit similar diffusion coefficients (Figure S8, Supporting Information).

However, in contrast to glycerol mixture, the nanoscopic viscosity probed by the beads exhibited a noticeable disparity compared to the macroscopic viscosity measured by the cone‐plate rheometer (Figure 5E). In poloxamer, the local viscosities determined by ILM were higher than the macroscopic viscosities. Several factors could account for this observation. First, it is important to consider the non‐Newtonian behavior of poloxamer 407, which, around 24 °C, is a binary mixture of monomers and micelles. Indeed, depending on the temperature and concentration, the polymer may self‐assemble generating micelles (≈20 nm in diameter), potentially interacting with the surface of particles. Furthermore, we confirmed here with beads of 100–300 nm that the viscosity is dependent on the scale at which it is measured, the viscosity being higher when measured by smaller particles. Moreover, the macroscopic viscosity was found even smaller than the viscosity experienced by the largest particles measured by ILM, revealing significant and larger long‐range interactions of the smallest beads with the micelles of the poloxamer.

We found here that ILM is a potent methodology for assessing the viscosity of poloxamer experienced by submicronic particles of similar size than EVs.

### 3.3 Size Distribution Measurement of EVs Dispersed in a Characterized Matrix of Poloxamer 407 (Measurement of *R*_h_ when *ɳ* Is Known)

In the previous section, we successfully determined the local viscosity of complex matrices by working with calibrated nanoparticles. Leveraging this measured parameter, we now investigate the diffusion patterns of bionanoparticles such as EVs in those complex environments.

**Figure 6:**
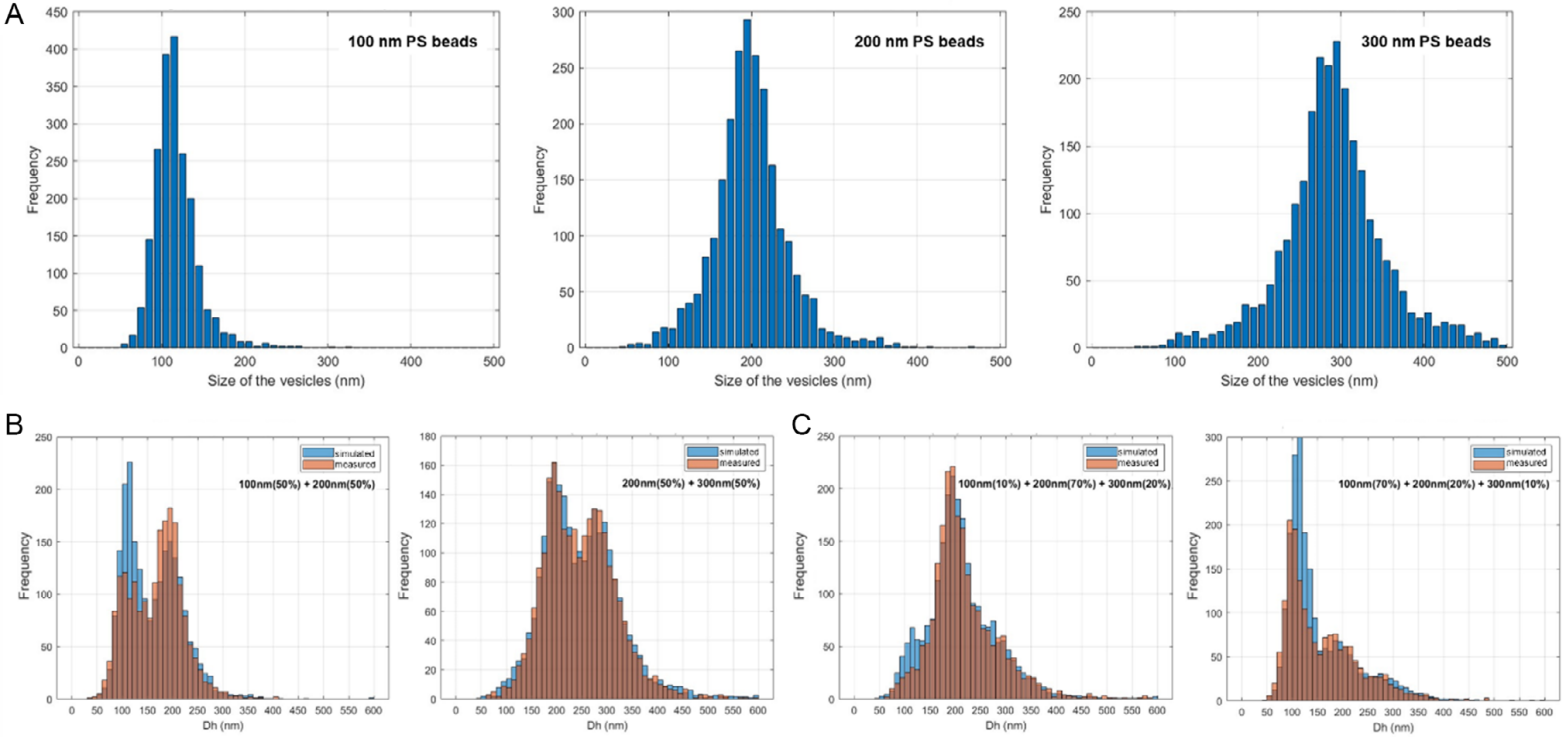
Validation of ILM for polydispersed sample characterization. A) Measured size distribution of monodispersed PS beads of 100, 200, and 300 nm beads. B) Simulated (in blue) and measured (in orange) size distribution of a binary mixtures of 100 and 200 nm (left, with *a* = 0.5, *b* = 0.5, *c* = 0) beads and 200 and 300 nm beads (right, with *a* = 0, *b* = 0.5, *c* = 0.5). C) Simulated (in blue) and measured (in orange) size distribution of ternary 100/200/300 nm bead mixtures (left: *a* = 0.1, *b* = 0.7, *c* = 0.2, right: *a* = 0.7, *b* = 0.2, *c* = 0.1).

EVs are highly heterogeneous in size, with size distributions broader than the calibration PS beads. In order to assess the instrument's measurement capabilities on a controlled polydispersed sample, we first performed ILM measurements on calibrated mixtures of PS beads with different sizes in PBS (**Figure** **6**). For each mix, the obtained size distribution (Figure 6B,C) was compared to the simulated distribution corresponding to the linear combination (Equation (6)) of the monodispersed experimental size distribution (Figure 6A) weighed by the mixture rates.

(6) $$H_{\text{simulated}}=a*H_{\text{100nm}}+b*H_{\text{200nm}}+c*H_{\text{300nm}}$$

where *a*, *b*, and *c* denote the respective percentages of beads of 100, 200, and 300 nm size respectively.

As shown in Figure 6B, the ILM system is efficient in detecting subpopulations of beads within mixtures comprising two or three distinct populations, with precise identification of each peak. This property is particularly crucial when studying EVs, given their inherent polydispersity in size. A smaller discordance was usually observed for the 100 nm‐beads population, whose size is close to the limit of detection of videodrop (80 nm).

In the second step, we combined both usages of ILM reported above. We have already quantified the local viscosity of a non‐Newtonian medium based on ILM analysis of particles of known size (Section 3.2). Leveraging the precharacterized matrix of poloxamer 407 using the calibration PS beads, we now employed ILM to study EVs derived from human adipose tissue stromal cells (hADSC‐derived EVs) and investigate their interactions with the poloxamer environment. The workflow of this study is outlined in detail in Figure 3. The validation of ILM for characterizing EVs in poloxamer 407 could not be performed by comparison to NTA, as NTA was unable to measure EVs in a viscous matrix. In the following, the dynamic of EVs was characterized in 7.5% poloxamer 407 formulation in comparison to the same EVs in aqueous suspension in PBS.

**Figure 7:**
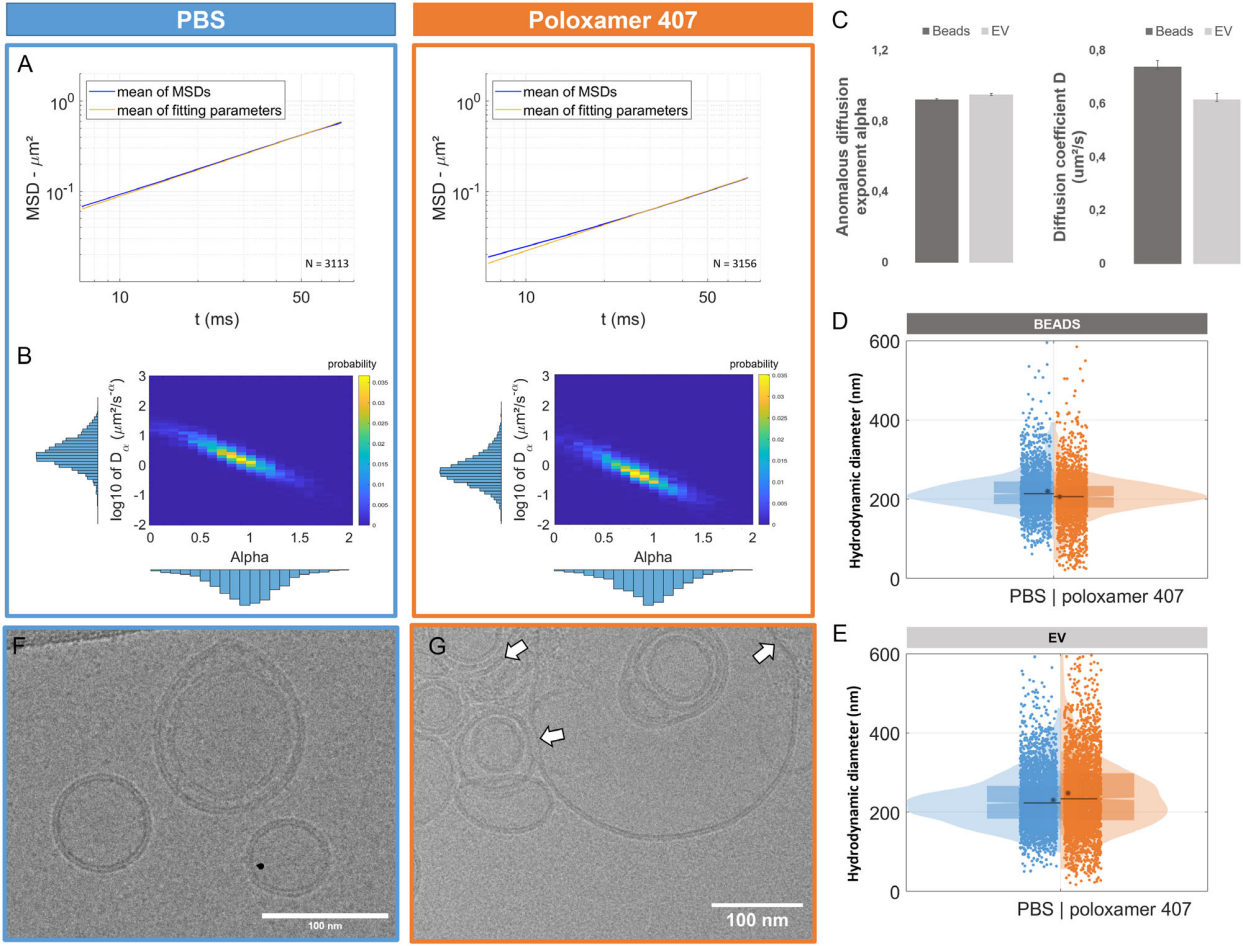
Characterization of EVs in 7.5% poloxamer 407 compared to PBS formulation. A) Representation of the mean of the fitted MSDs (±SEM) of EVs from hADSC cells in PBS (blue) and 7.5% poloxamer 407 (orange) and of the power law using the mean of the fitting parameters for each MSD in yellow. B) Representation of the distribution of the diffusion coefficient $D_{\alpha }$ as a function of the anomalous exponent *α* for the EVs in PBS (blue) and 7.5% poloxamer 407 (orange) (*N* = 3113 and 3156, respectively). C) Comparison of the anomalous diffusion exponent and the diffusion coefficient of 200 nm‐diameter beads and EVs in poloxamer 407 at 7.5%. D) Display of the size distribution of 200 nm‐diameter beads measured by ILM in PBS (blue) and 7.5% poloxamer 407 (orange). E) Display of the size distribution of EVs measured by ILM on PBS (blue) and 7.5% poloxamer 407 (orange). CryoEM images of EVs from hADSC cells F) in PBS (blue frame) and G) in 7.5% poloxamer 407 (orange frame). White arrows are pointing at identified areas of tension on the membrane of EVs.

As previously shown for the PS beads, we observed a good agreement between the geometric average of MSDs from each EV and the power law resulting from the average fitting parameters (**Figure** **7**A). hADSC‐derived EVs behave as freely diffusive particles both in PBS (with $D_{\alpha }=2.38+0.08\;/\;-0.03{\text{ μm}}^{2}s^{-1}$ and α=0.97 ±0.01) and in 7.5% poloxamer 407 (with $D_{\alpha }=0.62+0.02/-0.01{\text{ μm}}^{2}s^{-1}$ and α=0.95 ±0.01). The heatmaps corresponding to EVs in PBS (Figure 7B) closely resembled those of beads in water (Figure 2G), with an anomalous diffusion exponent centered around 1, in agreement with a purely diffusive regime. However, when embedded in poloxamer, the value of $D_{\alpha }$ for EVs is significantly reduced, indicating a decrease in diffusion rate. Despite this, the anomalous diffusion exponent remains close to 1, suggesting that EVs still undergo random walk diffusion in 7.5% poloxamer formulation (at an average temperature of 24 °C), albeit in a high‐viscosity medium.

Relying on an anomalous diffusion exponent close to 1, the size distribution of EVs could be derived according to the Stokes–Einstein equation in both PBS and poloxamer formulations (Figure 7E). The local viscosity was set to that of water for PBS formulation, while we leveraged the former analysis of local viscosity found in 7.5% poloxamer with beads of 200 nm (see Section 3.2) to derive the size distribution of biological particles (EVs) in the poloxamer formulation. This choice was motivated by the size distribution of EVs in PBS, which is centered around 200 nm resembling the size distribution measuring for bead of 200 nm, but with a larger distribution due to the intrinsic EV size heterogeneity. Interestingly, we observed in Figure 7E that the size distribution of EVs in 7.5% poloxamer formulation is significantly modified compared to PBS with an increase in both the mean and median size of the EVs along with a broader size distribution. This observation was consistent between the three independent productions of EVs as shown in Figure S9, Supporting Information. In comparison, the size distributions of 200 nm beads are comparable in PBS and 7.5% poloxamer formulation. Altogether, in the observed decrease in the diffusion coefficients of EVs in comparison to 200 nm beads in poloxamer formulation as well as the broadening and slight shift of the size distribution of EVs in poloxamer compared to PBS, both suggest significant interactions between the poloxamer and EVs that are not experienced by the PS beads.

To shed light on the potential mechanisms of interactions, CryoEM enabled direct visualization of EVs in both PBS and poloxamer matrices (Figure 7D). In both environments, the bilayer structure of EVs was clearly observed, demonstrating the presence of intact EVs. However, in poloxamer, concomitantly with intact EVs, we observed evidence of tension on some EVs, with apparition of broken membranes and release of proteins (Figure 7G and Figure S10, Supporting Information). This could indicate an interaction between the poloxamer 407 and EV membranes, leading to modifications in size distribution and, consequently, alterations in transport properties.

## 4 Discussion

While the use of EVs for regenerative medicine and drug delivery is rapidly expanding, there is an urgent need in developing new technologies for characterizing EVs in situ in complex formulation media. Specifically, the therapeutic potential and safety of EV formulations might strongly depend on their integrity, concentration, size, transport capability, conservation, and fate in those formulations. Moreover, considering the requirements of regulatory agencies for these emerging (nano)biotherapies, the definition of critical quality attributes is urgently expected. So far, most characterization methods, such as NTA or nanoflow cytometry, are being developed for EVs suspended in low‐viscosity medium, but are currently inapplicable for investigating in situ EVs in more complex and viscous environments. Nanorheology remains largely underexplored in the context of EV formulations, primarily due to the limited availability of instruments capable of accessing detailed information at this scale. The use of fluorescent probes in techniques like fluorescence correlation spectroscopy, which is primarily employed to study the surface domains of EVs, can also be adapted to determine size distributions.^[^ ^37^, ^38^ ^]^ However, a key limitation of this method is the requirement for particle labeling, which may alter the natural properties of the EVs and add complexity. The present work proposes a novel label‐free ILM‐based NTA strategy to gain insight into the dynamic behavior of EVs in clinically relevant formulations.

In a previous work, we used Poloxamer 407 (Pluronic F‐127), a thermosensitive gel (liquid at room temperature with a gel transition at body temperature), particularly interesting for the treatment of digestive fistulas.^[^ ^16^ ^]^ This previous study represented a first attempt to investigate the transport of EVs in Poloxamer 407 at high concentrations, to be close to the clinical 20% concentration, at which the viscosity is around 400 mPa.s (400 times the viscosity of water). An attempt to analyze EVs in a 20%, 10%, and 2% poloxamer 407 dispersion using NTA (LM 10, Malvern Panalytical) was performed in this qualitative study. As it could be expected, no displacement of EVs could be observed at 20% concentration. At 10% concentration, the supposedly Brownian motion of EVs was partially restored yet reduced compared to EVs in 2% poloxamer 407 or in PBS control condition. These results indicated that the effect of the poloxamer formulation was reverted by the matrix dilution. However, this first attempt only provided qualitative evaluation of EV mobility in the formulation.

In the present article, we go far beyond a qualitative investigation of EVs in complex formulation and propose the first quantitative analysis of EV spontaneous thermal motion in Poloxamer 407 at different concentrations (up to 7.5%) in comparison to PS nanobeads. We used the ILM instrument, an alternative ILM‐based NTA technology, to record images, but we bypassed automated image treatment and analysis of the commercial setup, in order to: 1) record in situ the trajectories of several thousand of single EVs in complex fluids with nanoscale resolution and analyze their MSD, 2) investigate the behavior of the EV motion and potential deviation from Brownian motion, 3) measure the viscosity at the nanoscale for Newtonian and non‐Newtonian fluids via calibration beads of known size, 4) use this viscosity data to determine the size distribution of Brownian EVs in non‐Newtonian poloxamer 407 formulation, and 5) highlight the interactions between EVs and the matrix of poloxamer 407.

Macroscopic viscosity of poloxamer 407 can be measured using standard rheology, as reported by our group^[^ ^15^ ^]^ and others.^[^ ^39^ ^]^ However, as poloxamer 407 is a non‐Newtonian fluid, we wanted to investigate the viscosity that is experienced by nanobioparticles such as EVs at the nanoscale and shed light on their potential interactions with the poloxamer matrix. As expected for non‐Newtonian fluid, for which viscosity depends on the global or local shear rates, slight differences were observed between the local viscosity measured by 100 nm versus 200 nm versus 300 nm PS probe beads as well as with the global viscosity measured by the cone plane rheometer. Differences between the macroscopic and the nanoscopic viscosity values reinforced the fact that it is important to measure the viscosity at the same size range than the particle of interest. This is particularly relevant for EV therapeutic nanovectors that might interact with the matrix used for their delivery in the body, especially for local administration. Here we demonstrated this point for a thermosensitive poloxamer that showed impressive generative properties when delivering stem cell EVs in fistulas.

Understanding the interactions between EVs and its surrounding matrix is crucial for advancing innovative therapeutic strategies, as these interactions govern the stability, transport, and delivery of cargo. The influence of the matrix's viscosity on lipidic nanovectors has been extensively investigated. For instance, Bochot et al. demonstrated that poloxamer 407 can disrupt lipidic membranes,^[^ ^40^ ^]^ leading to the formation of pores or fractures that facilitate cargo release, which is in agreement with our cryoEM observations. Interestingly, the addition of lipidic nanoparticles does not substantially alter the macroscopic viscosity of the matrix. A recent study has shed light on how the interplay between the matrix and liposomes can selectively influence subpopulations, resulting in distinct behavioral and transport properties.^[^ ^41^ ^]^ They emphasize how these interactions, and their resulting effect, strongly hinge on the specific characteristics of the bionanoparticles being examined. However there is a need for accurately predicting particle movement in situ in viscoelastic environments, necessitating a comprehensive analysis of trajectories.^[^ ^28^, ^41^ ^]^

Controlling rheological properties is crucial for gaining deeper insight into the physical characteristics, structure, stability, and drug release profiles of pharmaceutical formulations.^[^ ^42^, ^43^, ^44^ ^]^ Understanding the viscoelastic properties at the particle scale is particularly important in a wide range of biomedical and pharmaceutical fields,^[^ ^45^ ^]^ as these properties influence the performance and behavior of the formulation in various environments. However, accurately measuring the viscoelastic behavior of polymers, especially near surfaces or under confinement, presents a significant experimental challenge due to the complexities of nanoscale interactions.^[^ ^46^ ^]^ The manufacturing process and quality control for biotherapies like EVs must adhere to the guidelines required for marketing authorization (Common Technical Document, Module 3, ICH Topic M4Q).^[^ ^47^, ^48^ ^]^ As stipulated by the EMA/CHM/BWP/534898/2008,^[^ ^49^ ^]^ rigorous quality control is required for both the drug substance (active substance, in our case EVs) and the drug product (finished product, in our case EVs in poloxamer), with mandatory specifications for the following quality attributes: quantity, identity, purity, and microbiological quality, completed by biological activity tests. This article addresses the challenges of performing quality control for the drug product when EVs are in a complex viscous medium. More specifically, it proposes a clear analytical workflow for assessing the quality attributes of quantity and identity, particularly in terms of size. The insights provided herein are crucial for non‐Newtonian viscous formulations, for which analytical methods used for the quality control of the drug substance may no longer provide reliable data.

Here, by analyzing the MSD of thousands of particles individually and by comparing their behavior to the one of nonbiological particles in the same media, we were able to identify the type of motion without assumption of Brownian behavior. It is only when we validated the Brownian motion (α≈1), that we can use the size distribution deduced from the MSD through the Stokes–Einstein equation and quantify the impact of the poloxamer on the size of EVs. When examining EVs in the poloxamer 407, we observed distinct forces acting on the biological particles that could lead to size distribution distortion. Additionally, as EVs are deformable nano‐objects, their size and shape could be potentially altered by the poloxamer, as it was suggested by cryoEM images. Furthermore, the release of the proteins derived from EVs may contribute to changes in the local viscosity of the poloxamer 407, impacting even further the transport behavior of both EVs and other particles (like protein aggregates) within the matrix. This effect would then depend on the composition of the EV membrane and its integrity. Poloxamer 407 (also known as Pluronic block copolymers) has amphiphilic properties that enable it to act like a surfactant, with the ability to interact with and potentially destabilize biological membranes.^[^ ^50^ ^]^ A different impact of poloxamer on EVs could happen at higher concentration of poloxamer, as it was described in this same paper that the formation of micelles diminishes the capability of poloxamer 407 to affect cellular membranes.

The utilization of ILM in this study has provided valuable insights into the interplay between nanoparticle transport and nanorheology within complex fluid. Using ILM, we were able to not only measure size distribution, as expected by the instrument, but also to comprehensively highlight interactions of nanoparticles with their environment in a comprehensive manner. This capability is due to the setup's geometry, which differs from instruments requiring fluidic sample insertion. The novelty of our approach lies in exploring the relationship between biological nanoparticles and the physical properties of their surrounding environment. It represents a significant advancement in the characterization of nanoparticles, particularly in complex and viscous media, which are often encountered in the context of gel‐based EV formulations. The results obtained from this study underscore the importance of considering both intrinsic nanoparticle properties and the rheological characteristics of the surrounding matrix when formulating EVs for therapeutic purposes. Unlike traditional microscopy techniques, ILM offers several advantages, including high sensitivity, real‐time imaging capabilities, and the ability to work in complex media with relatively high viscosity. This makes ILM particularly well‐suited for studying the interactions between nanoparticles and their surrounding environment in biologically relevant contexts. The methodology proposed in this manuscript could be applied to any system with limitations regarding: (1) interferometric contrast: the EVs or probes need to have a refractive index different from the medium or its constituents and (2) motion detection and trajectory: to be detected by the system, the EVs or probes must exhibit displacements higher than the pixel size (56.7 nm) between two images (7.15 s) and be followed for at least ten images.

Overall, our findings shed light on the intricate interplay between bioparticles and their surrounding matrix. The integration of ILM for rheological analysis represents a promising approach for investigating quantitatively the nano(bio)particle transport in complex environments. By characterizing statistically the individual trajectory of thousands of EVs simultaneously, we pave the way for the development of quality control attributes and more effective bionanotherapies. The advancement of biotherapies based on EVs is currently hindered by two major challenges: the absence of standardized methods for material production and the lack of reliable quality control processes. To propel this field forward, the development of innovative methodologies is crucial. This work is firmly rooted in this philosophy, aiming to address these challenges and contribute to the progress of EV‐based biotherapies

## 5 Conclusion

In conclusion, the utilization of ILM presents a promising avenue for the characterization of nanoparticles, including EVs, especially within complex matrices of high viscosity. We demonstrate the advantages of ILM, such as its ability to measure particle concentration, to track single‐particle trajectory, and to infer particle size distribution with calibration‐like procedures and validation against known standards. Furthermore, ILM can provide quantitative information about the nanorheological behavior of non‐Newtonian fluids. Overall, our study highlights the potential of ILM to provide insights into the interactions between nanobioparticles and their surrounding environment, representing a valuable quality control test for EV hydrogel formulations intended to clinical use. Moving forward, the continued development and refinement of ILM techniques hold promises for advancing our understanding of nanoparticle physical behavior and facilitating their utilization in diverse biomedical contexts.

## Conflict of Interest

F.G. and A.K.A.S. are cofounders and shareholders of the spin‐off Evora Biosciences. Additionally, A.K.A.S. is a co‐founder of the spin‐off EVerZom. F.G. and A.K.A.S. are also shareholders of the spin‐off EVerZom.

## Author Contributions

**Lucile Alexandre**: Investigation (equal); Methodology (equal); Software (equal); Writing—original draft (equal). **Anastasiia Dubrova**: Investigation (equal). **Aruna Kunduru**: Investigation (equal). **Estelle Surply**: Investigation (equal). **Christopher Ribes**: Investigation (equal). **Imane Boucenna**: Investigation (equal). **Florence Gazeau**: Conceptualization (lead); Writing—review and editing (lead). **Amanda K. A. Silva**: Conceptualization (equal); Methodology (equal); Supervision (equal); Writing—original draft (equal). **Stéphanie Mangenot**: Conceptualization (equal); Investigation (equal); Methodology (equal); Supervision (equal); Writing—original draft (equal). **Kelly Aubertin**: Conceptualization (lead); Investigation (equal); Methodology (lead); Supervision (lead); Writing—original draft (equal).

## Supporting information

Supplementary Material

## Data Availability

The data that support the findings of this study are available from the corresponding author upon reasonable request.
