# Supplementary material for: Investigating Extracellular Vesicles in Viscous Formulations: Interplay of Nanoparticle Tracking and Nanorheology via Interferometric Light Microscopy
Source: Small Sci. 2024 Nov 18;5(1):2400319. doi: 10.1002/smsc.202400319 (PMC11935215; doi:10.1002/smsc.202400319)
Supplement: Supplementary file 1 — Supplementary Material [file SMSC-5-2400319-s001.pdf]

SUPPORTING INFORMATION

Investigating Extracellular Vesicles in Viscous Formulations: Interplay of Nanoparticle Tracking and Nanorheology via Interferometric Light Microscopy

Lucile Alexandre[1,*], Anastasiia Dubrova[1], Aruna Kunduru[1], Imane Boucenna[1], Florence Gazeau[1], Amanda K. A. Silva[1], Stéphanie Mangenot[1], Kelly Aubertin[1,*]

[1] Université Paris Cité, MSC, CNRS, 45 rue des Saints-Pères 75006 PARIS

* Corresponding authors: kelly.aubertin@u-paris.fr, lucile.alexandre@u-paris.fr

Table des matières

Supplementary Table S1 2
Supplementary Figure S1 3
Supplementary Figure S2 4
Supplementary Figure S3 5
Supplementary Figure S4 6
Supplementary Figure S5 7
Supplementary Figure S6 8
Supplementary Figure S7 9
Supplementary Figure S8 9
Supplementary Figure S9 10
Supplementary Table S2 12

Supplementary Table S1: Introduction to the vocabulary used in the article

Notions	Explanations
**Nanorheology**	**Nanorheology** is the study of the flow and deformation properties of materials at the nanometer scale (10^{-9} meters), with a particular focus on the mechanical properties, such as viscosity. At this scale, factors like molecular interactions, surface forces, and confinement effects can significantly alter how materials flow and deform, requiring specialized techniques and instruments for accurate measurement. In recent years, thanks to new technological development, nanorheology has garnered considerable attention due to its significance in fields such as biology, medicine, and materials science.
**Mean Squared Displacement (MSD)**<br>**Subdiffusive motion**<br>**Superdiffusive motion**	The **Mean Squared Displacement (MSD)** is a statistical measure that quantifies the distance travelled relative to a reference position over time. It represents the space explored by the particle within a certain time frame. MSD is widely used to characterize the type of movement a particle undergoes and its interactions with the environment during its motion. For **normal diffusion**, MSD increases linearly with time. However, when there are movement restrictions, MSD grows more slowly than linearly, indicating **subdiffusive motion**. On the other hand, if there is a preferential or forced direction, MSD increases faster than linearly, indicating **superdiffusive motion** (cf figure 1).
**Diffusion coefficient (D)**<br>**Anomalous diffusion exponent (α)**	MSD can be modelled using an equation with two key coefficients: the **diffusion coefficient (D)** and the **anomalous diffusion exponent (α)**. The diffusion coefficient (D) is related to the rate at which the particle moves, while the anomalous diffusion exponent (α) describes the extent to which the motion deviates from normal diffusion. Specifically, $\alpha=1$ corresponds to normal diffusion, $\alpha<1$ indicates **subdiffusion**, and $\alpha>1$ indicates **superdiffusion**
**Viscosity**<br>**Newtonian fluids**<br>**Non-Newtonian fluids**	**Viscosity** refers to the internal friction within a fluid, measured by the force per unit area that resists uniform flow. It quantifies a fluid's resistance to flow or deformation when subjected to an applied force. **Newtonian fluids**, such as water or glycerol, have a constant viscosity that remains unchanged regardless of the rate of applied shear stress, meaning their resistance to flow remains unchanged whether they are stirred slowly or rapidly. In contrast, **non-Newtonian fluids**, like poloxamer 407, exhibit viscosity that changes based on the applied stress or shear. Their flow behaviour can vary due to factors such as time, temperature, or the rate at which force is applied, leading to more complex flow characteristics

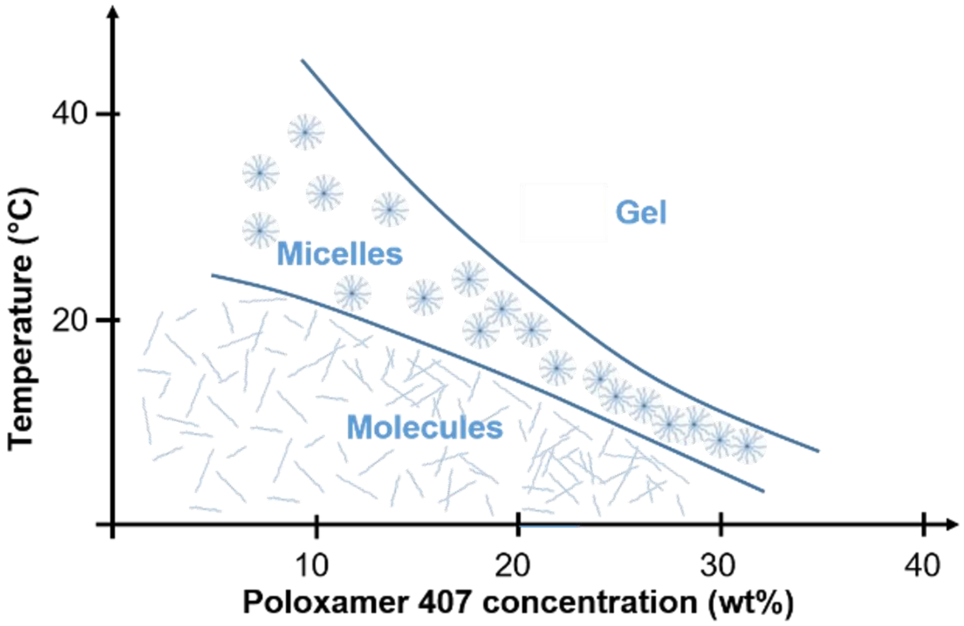

Supplementary Figure S1: Phase diagram of poloxamer 407, showing 3 different phases: monomers, micelles, gel.

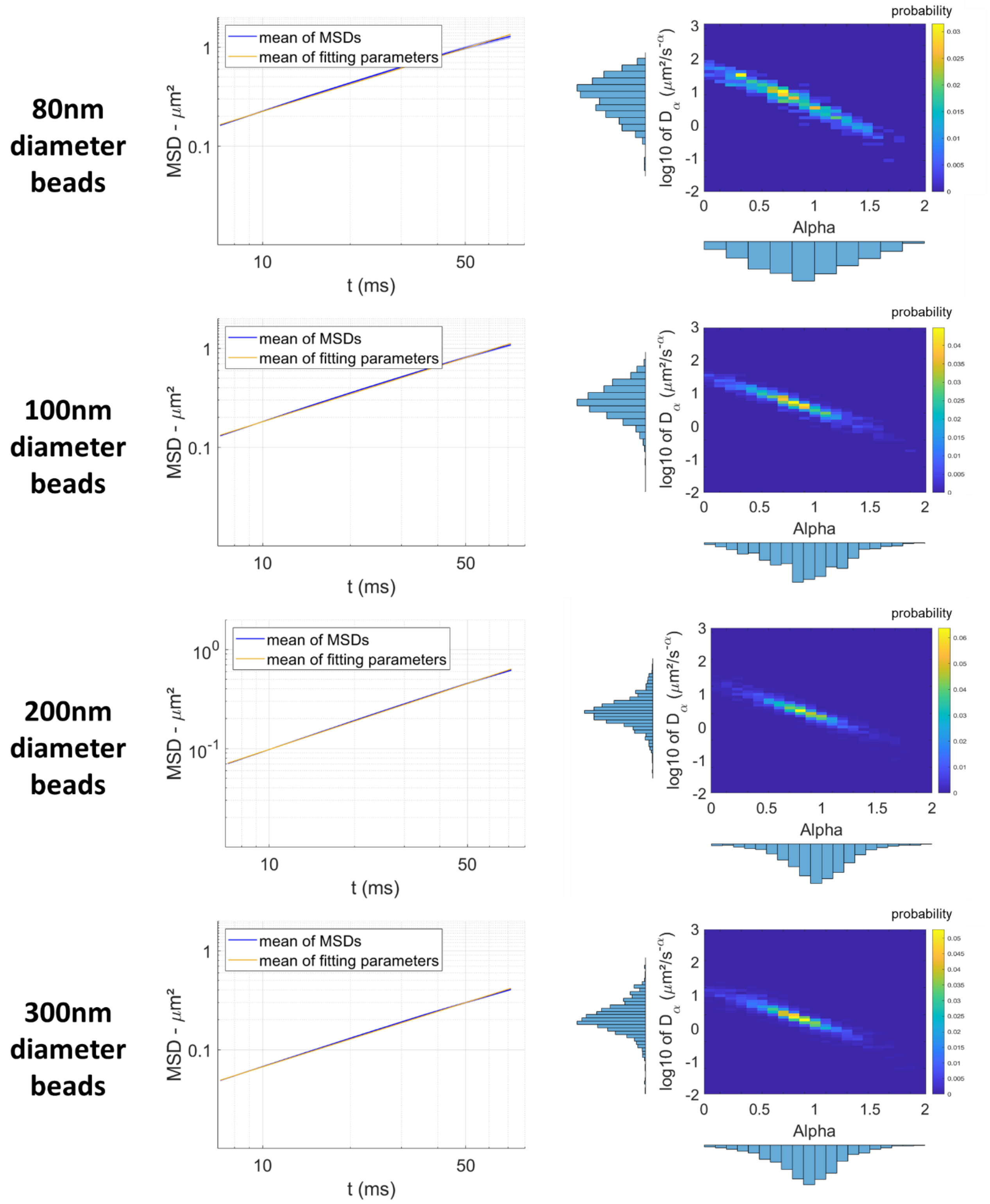

Supplementary Figure S2: Geometric average MSD for 80nm, 100 nm, 200 nm and 300 nm beads in PBS and the correspondent heatmaps of diffusion coefficients as a function of the anomalous diffusion exponents obtained for each MSD.

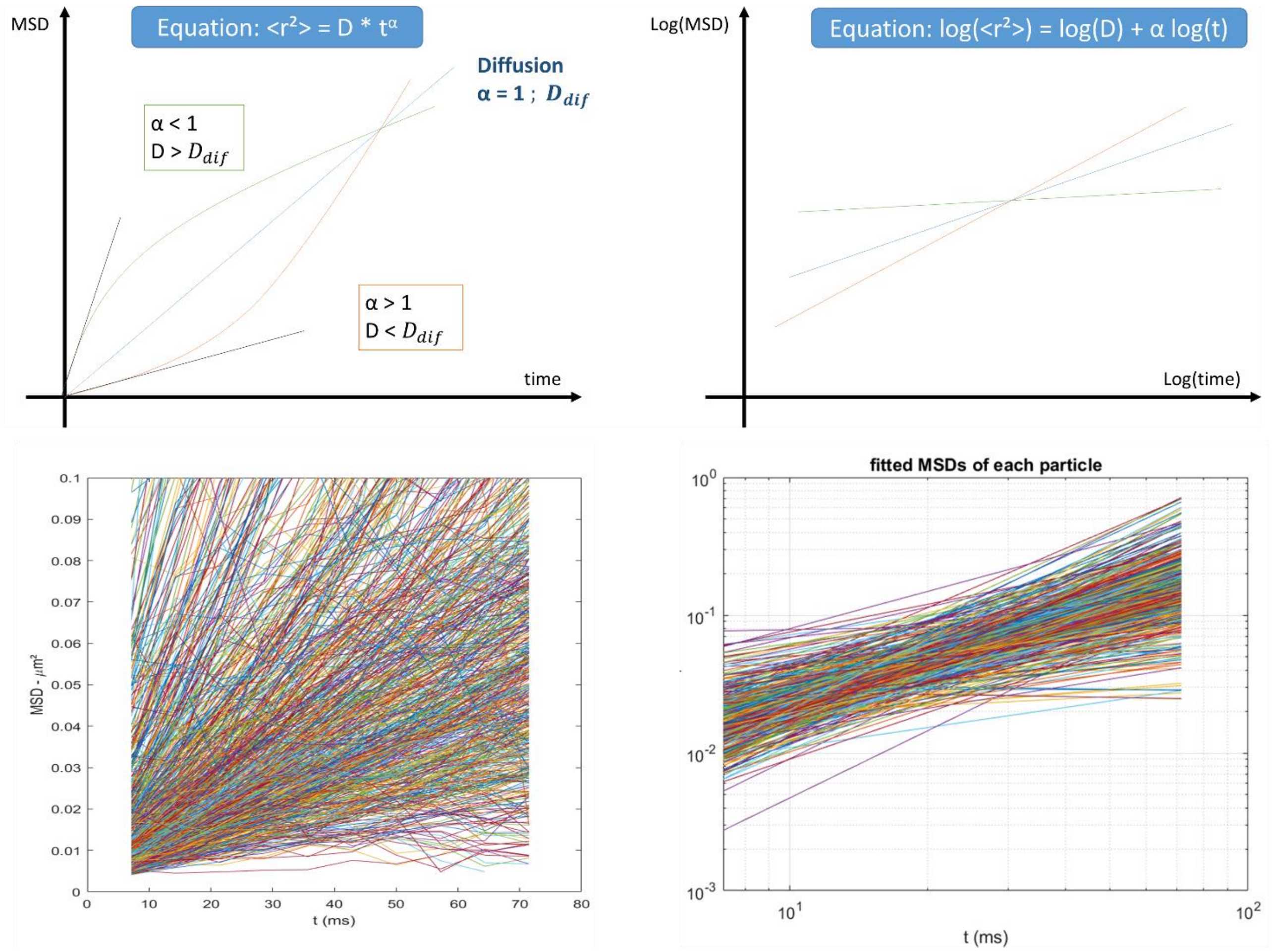

Supplementary Figure S3: graphical simulation of MSD (top) and experimental MSD fits from 200nm beads (N=500) in PBS (bottom) potentially explaining the correlation between the diffusion coefficient D_α and the anomalous diffusion exponent α (left: in linear scale, right: in log scale).

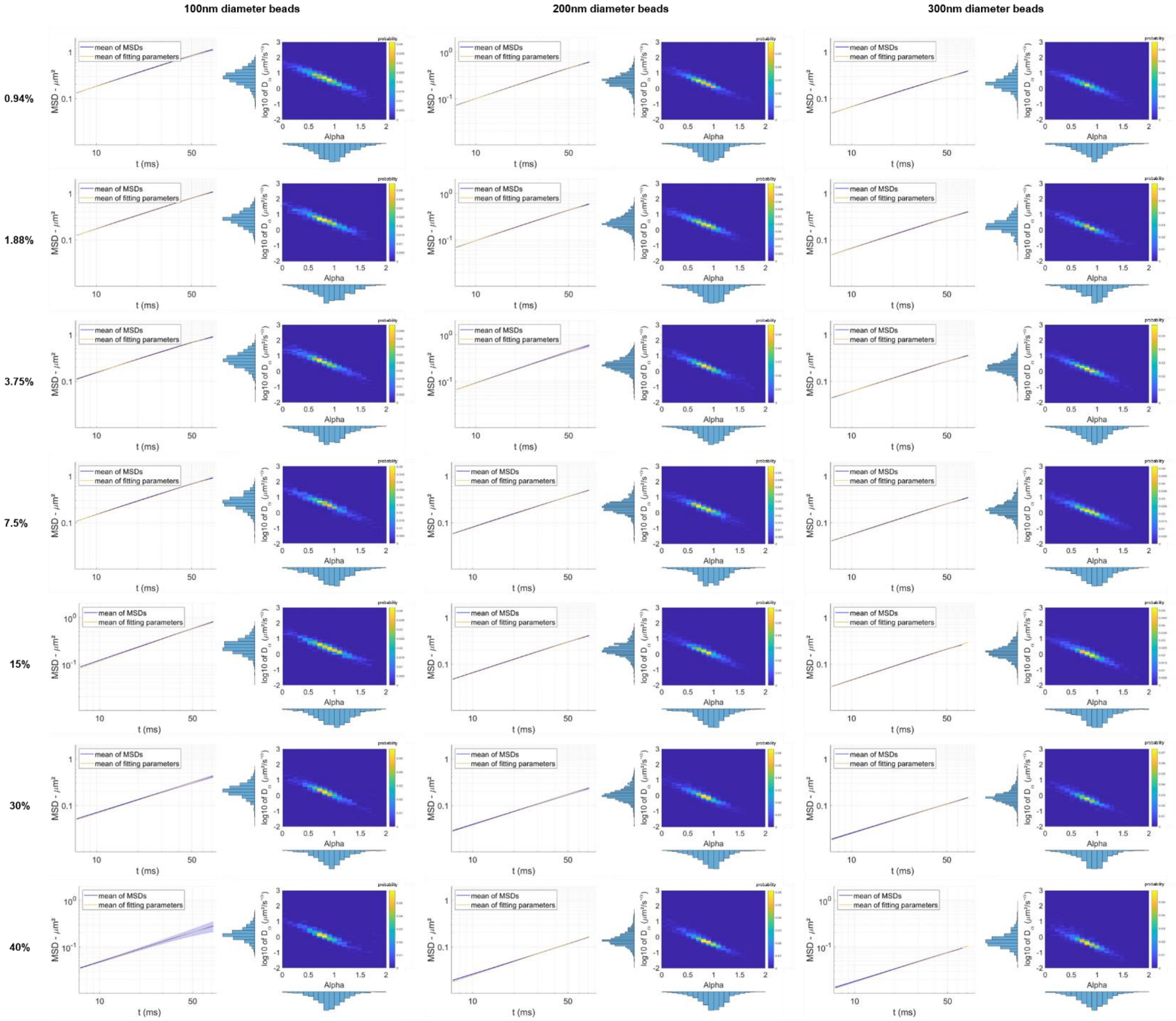

Supplementary Figure S4: Geometric average MSD +/- SEM for 100 nm, 200 nm, 300 nm PS beads embedded in 0.94%, 1.88%, 3.75%, 7.5%, 15%, 30% and 40% of glycerol and the corresponding heatmaps of diffusion coefficients as a function of the anomalous diffusion exponents obtained for each MSD.

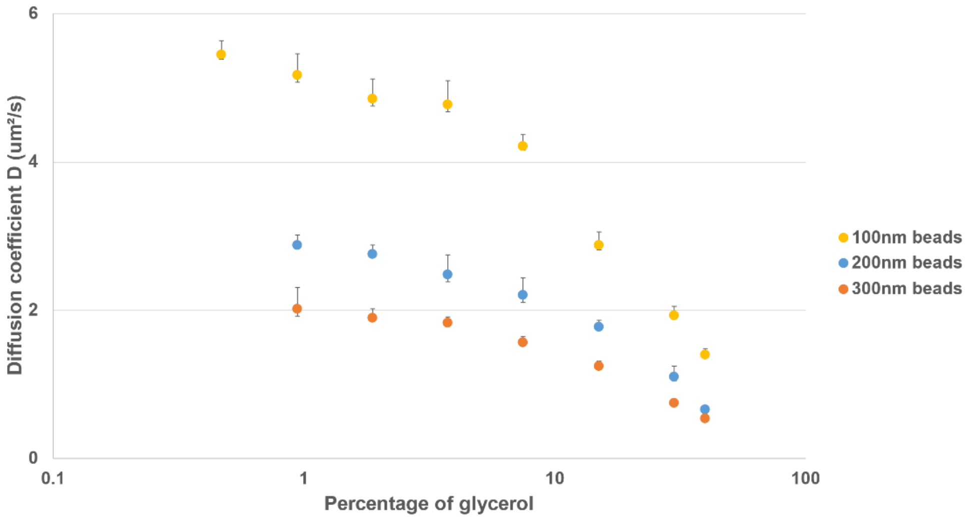

Supplementary Figure S5: Diffusion coefficients of 100 nm, 200 nm and 300 nm PS beads as a function of the percentage of glycerol.

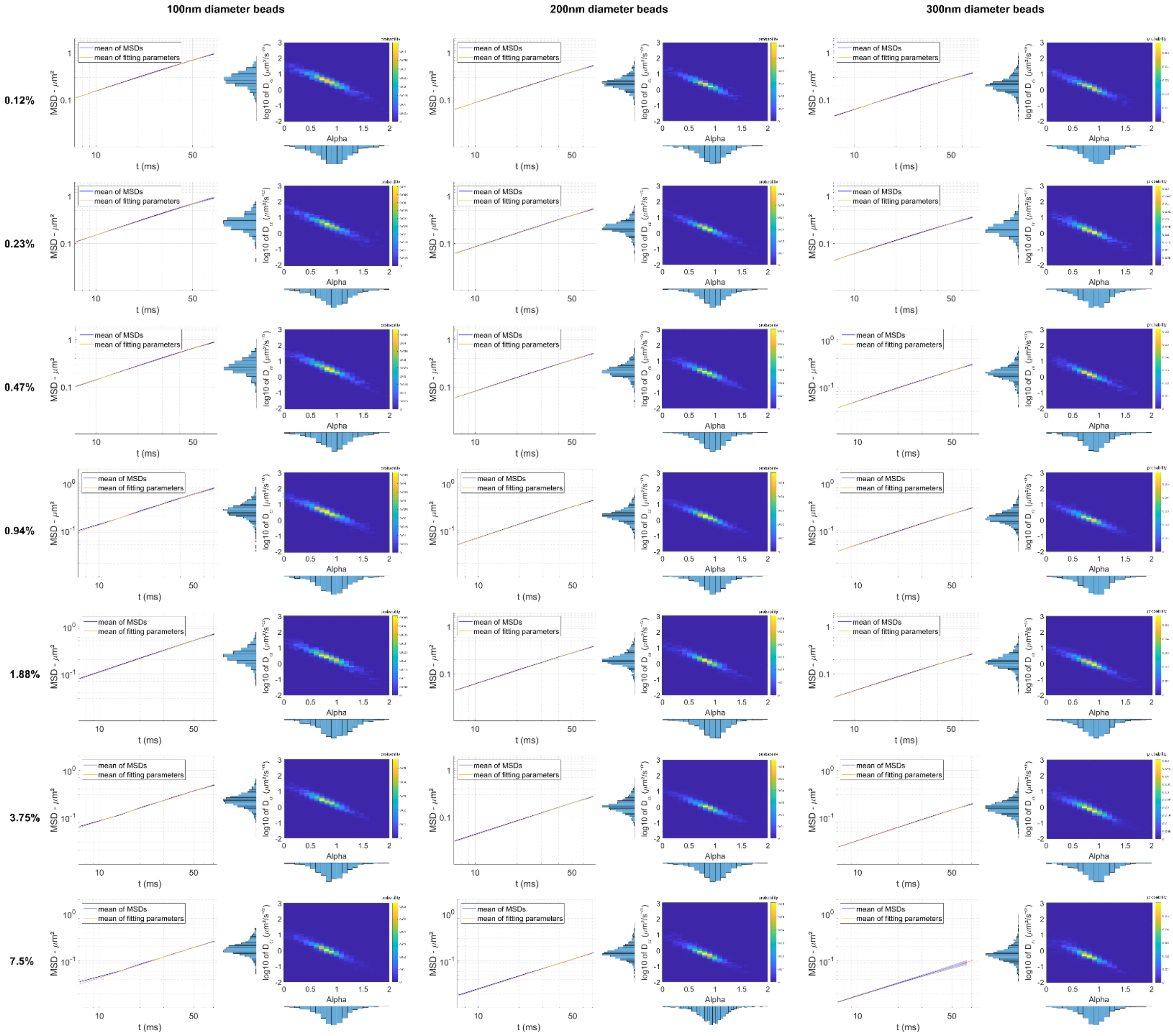

Supplementary Figure S6: Geometric average MSD +/- SEM for 100 nm, 200 nm, 300 nm PS beads embedded in 0.12%, 0.23%, 0.47%, 0.94%, 1.88%, 3.75%, 7.5% of poloxamer 407 and the corresponding heatmaps of diffusion coefficients as a function of the anomalous diffusion exponents obtained for each MSD.

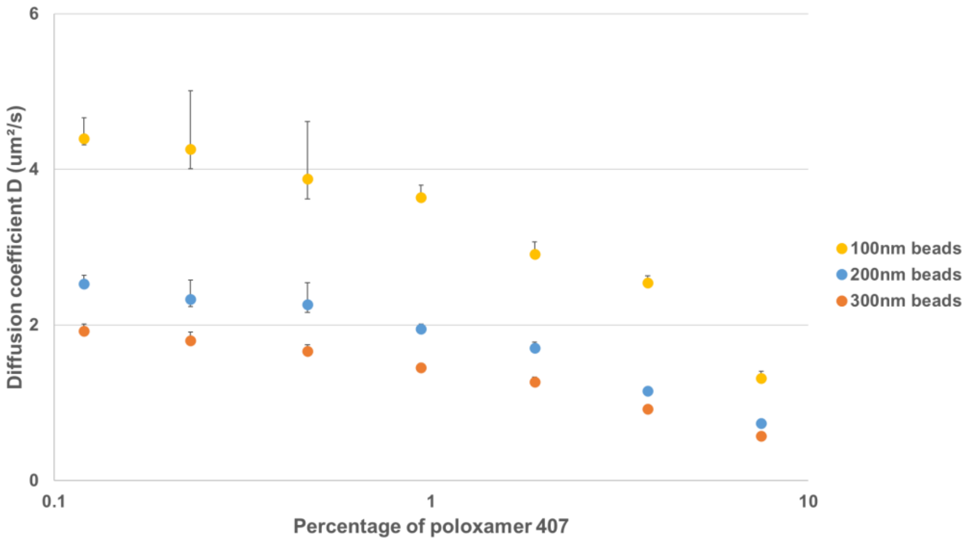

Supplementary Figure S7: Diffusion coefficients of 100 nm, 200 nm and 300 nm PS beads as a function of the percentage of poloxamer 407.

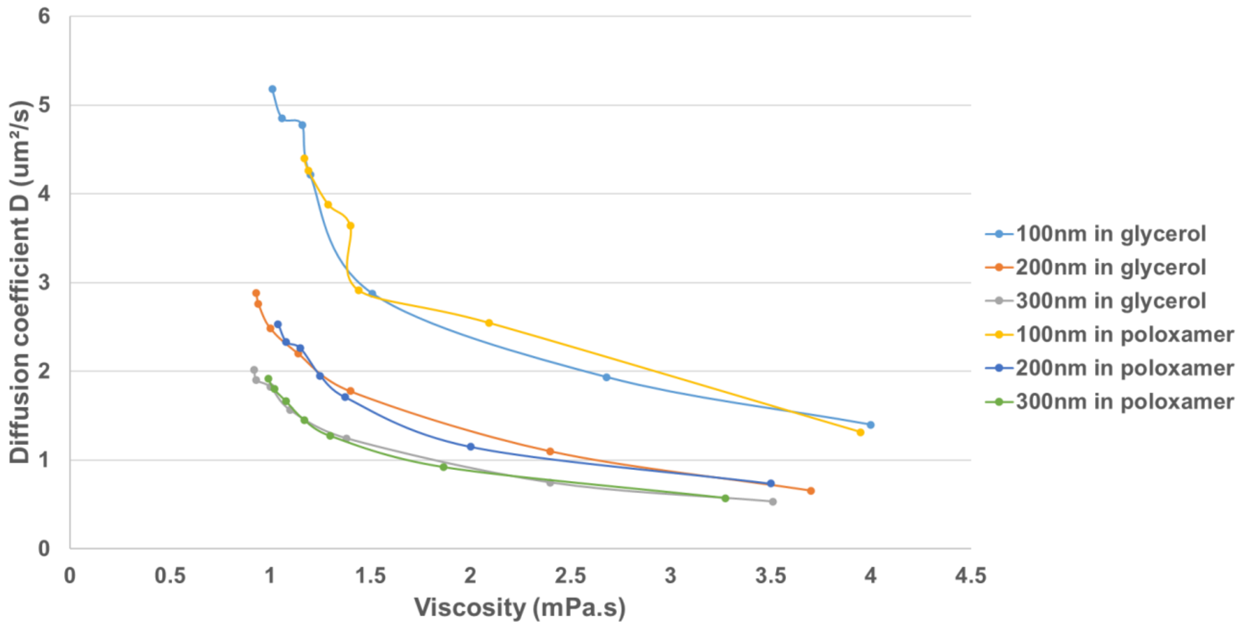

Supplementary Figure S8: Diffusion coefficients as a function of the viscosity measured by 100, 200 and 300 nm PS-beads in glycerol or poloxamer 407 at various concentrations.

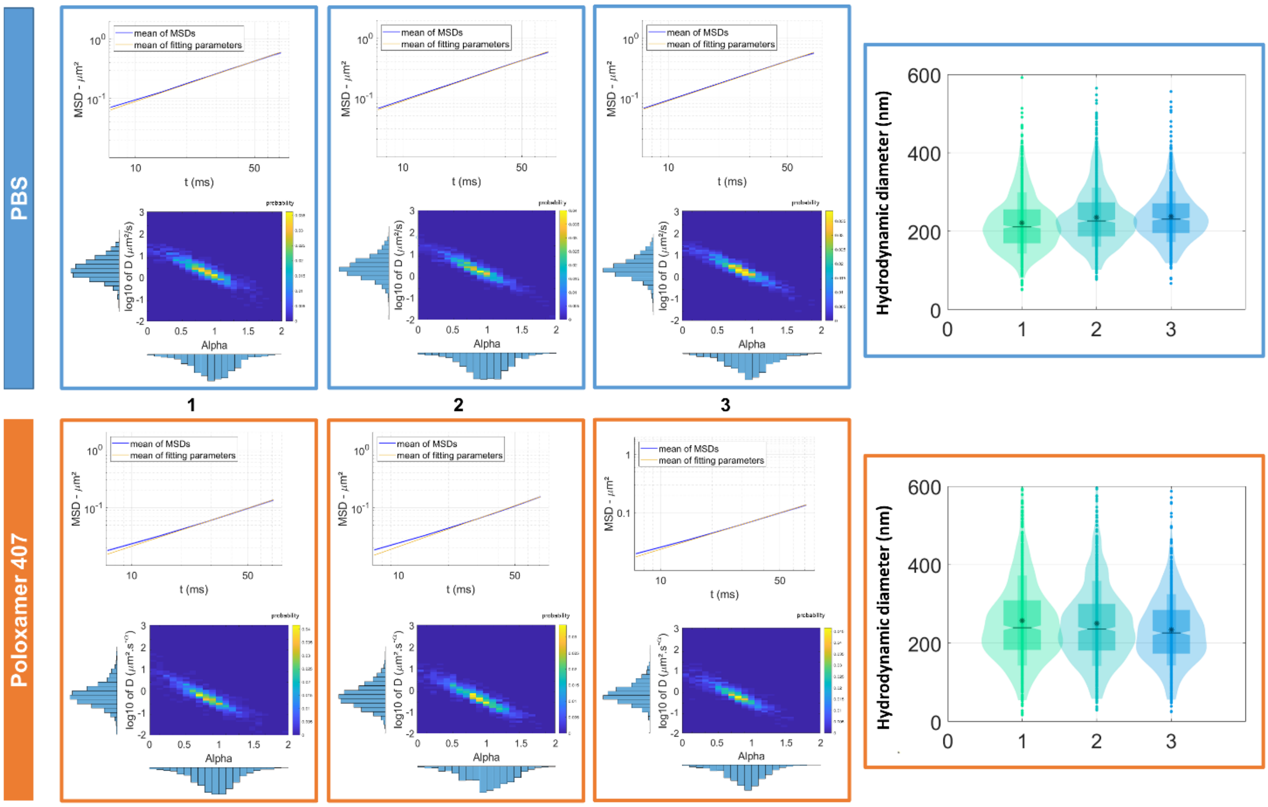

Supplementary Figure S9: Geometric average MSD +/- SEM from 3 different hASC-EV productions (1-3) in PBS or in 7.5% poloxamer 407, the corresponding heatmaps of diffusion coefficients as a function of the anomalous diffusion exponents obtained and the corresponding hydrodynamic diameter distributions.

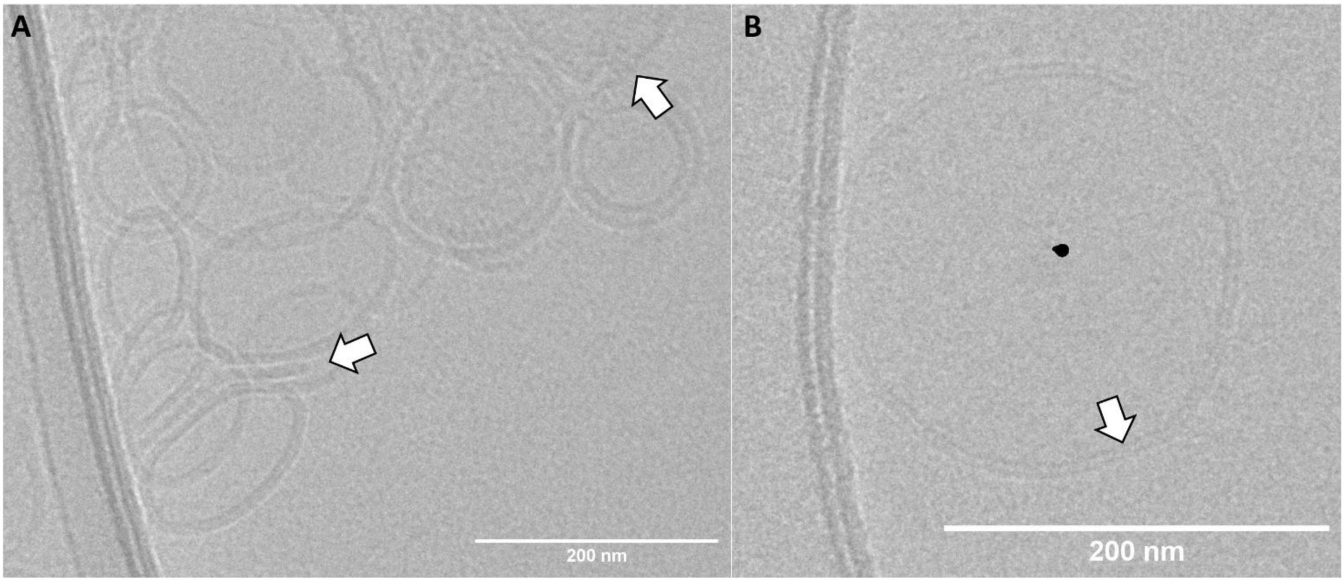

Supplementary Figure S10: Display of the tension on the membrane of EVs from hADSC cells in 7.5% poloxamer 407, observed by cryoEM. Tensions were observed in both A) populations of EVs and B) single EVs. White arrows indicate areas of tension on the EV membranes, highlighting membrane disruptions, protein aggregates, and deformations associated with increased membrane rigidity

Supplementary Table S2: recapitulative table of the average values +/- SEM of the diffusion coefficient and the anomalous exponent for 100, 200 and 300 nm PS-beads in Glycerol or Poloxamer 407 at various concentrations.

	Glycerol 40%					
	100nm beads		200nm beads		300nm beads	
	α	D (µm²/ms)	α	D (µm²/ms)	α	D (µm²/ms)
Values	0.92175041	0.00139702	0.96922325	0.00065783	0.91280099	0.00053211
SEM-	0.01115719	3.168E-05	0.00850336	1.2101E-05	0.00867684	3.0122E-05
SEM+		7.8104E-05		3.2705E-05		9.0528E-05
	Glycerol 30%					
	100nm beads		200nm beads		300nm beads	
	α	D (µm²/ms)	α	D (µm²/ms)	α	D (µm²/ms)
Values	0.94129084	0.00193367	0.93631216	0.00109957	0.91772934	0.000747
SEM-	0.01069026	4.2119E-05	0.00794872	5.4549E-05	0.00853297	1.3529E-05
SEM+		0.00011285		0.00014031		3.8126E-05
	Glycerol 15%					
	100nm beads		200nm beads		300nm beads	
	α	D (µm²/ms)	α	D (µm²/ms)	α	D (µm²/ms)
Values	1.00312269	0.00287795	0.95529591	0.00177631	0.954135	0.00124554
SEM-	0.01150175	6.2028E-05	0.0088727	3.191E-05	0.00902208	2.2453E-05
SEM+		0.00017785		8.9943E-05		6.6591E-05
	Glycerol 7.5%					
	100nm beads		200nm beads		300nm beads	
	α	D (µm²/ms)	α	D (µm²/ms)	α	D (µm²/ms)
Values	0.94465984	0.00421255	0.95161564	0.00219968	0.94633219	0.00156311
SEM-	0.01123129	9.0353E-05	0.00797899	9.0153E-05	0.00848771	2.5778E-05
SEM+		0.00026797		0.00024233		7.3807E-05
	Glycerol 3.75%					
	100nm beads		200nm beads		300nm beads	
	α	D (µm²/ms)	α	D (µm²/ms)	α	D (µm²/ms)
Values	0.91177669	0.00477814	0.9622513	0.00248385	0.91864502	0.00182419
SEM-	0.01128073	0.00010235	0.0080295	9.683E-05	0.00830547	3.0075E-05
SEM+		0.0003196		0.00026201		7.9825E-05
	Glycerol 1.88%					
	100nm beads		200nm beads		300nm beads	
	α	D (µm²/ms)	α	D (µm²/ms)	α	D (µm²/ms)
Values	0.94704131	0.00485214	0.93845036	0.00276238	0.93550844	0.0019004
SEM-	0.01051374	9.6999E-05	0.00803768	4.3377E-05	0.01037961	3.9192E-05
SEM+		0.00026485		0.00011933		0.00011215
	Glycerol 0.94%					
	100nm beads		200nm beads		300nm beads	
	α	D (µm²/ms)	α	D (µm²/ms)	α	D (µm²/ms)
Values	0.94368053	0.00517913	0.93458852	0.00288185	0.9146794	0.00201399
SEM-	0.01005866	9.8136E-05	0.00857115	4.9426E-05	0.00923924	9.5221E-05
SEM+		0.00028447		0.00013041		0.0002936

	Poloxamer 407 7.5%					
	100nm beads		200nm beads		300nm beads	
	α	D (µm²/ms)	α	D (µm²/ms)	α	D (µm²/ms)
Values	0.91977533	0.00131341	0.92190119	0.000737835	0.88968178	0.000572688
SEM-	0.00641653	3.5901E-05	0.00569752	9.13508E-06	0.00648878	7.80051E-06
SEM+		9.017E-05		2.40654E-05		2.14709E-05
	Poloxamer 3.75%					
	100nm beads		200nm beads		300nm beads	
	α	D (µm²/ms)	α	D (µm²/ms)	α	D (µm²/ms)
Values	0.9111874	0.0025453	0.96437469	0.001151781	0.93415628	0.000921894
SEM-	0.00636217	3.3103E-05	0.00600014	1.38539E-05	0.00633094	1.19513E-05
SEM+		8.7563E-05		3.57737E-05		3.28589E-05
	Poloxamer 1.88%					
	100nm beads		200nm beads		300nm beads	
	α	D (µm²/ms)	α	D (µm²/ms)	α	D (µm²/ms)
Values	0.96529197	0.00291118	0.94662706	0.001707539	0.93095122	0.001272055
SEM-	0.00994118	5.6595E-05	0.00811108	2.81368E-05	0.00816574	2.11582E-05
SEM+		0.00015801		7.58797E-05		5.79164E-05
	Poloxamer 0.94%					
	100nm beads		200nm beads		300nm beads	
	α	D (µm²/ms)	α	D (µm²/ms)	α	D (µm²/ms)
Values	0.93864106	0.003643	0.94747853	0.001951572	0.93073094	0.001452112
SEM-	0.0101672	5.4877E-05	0.0060523	2.36938E-05	0.0064102	1.8827E-05
SEM+		0.00015384		6.32816E-05		5.53041E-05
	Poloxamer 0.47%					
	100nm beads		200nm beads		300nm beads	
	α	D (µm²/ms)	α	D (µm²/ms)	α	D (µm²/ms)
Values	0.9275997	0.00387842	0.95078035	0.002262135	0.92770966	0.001661423
SEM-	0.00726175	0.00025556	0.00819675	0.000101599	0.00849547	2.81939E-05
SEM+		0.00073745		0.000284717		8.19923E-05
	Poloxamer 0.23%					
	100nm beads		200nm beads		300nm beads	
	α	D (µm²/ms)	α	D (µm²/ms)	α	D (µm²/ms)
Values	0.94474988	0.00426409	0.95492701	0.002330498	0.9222471	0.00179954
SEM-	0.00998979	0.00025667	0.00787405	9.45012E-05	0.00922101	3.31518E-05
SEM+		0.00074576		0.000248015		0.000108674
	Poloxamer 0.12%					
	100nm beads		200nm beads		300nm beads	
	α	D (µm²/ms)	α	D (µm²/ms)	α	D (µm²/ms)
Values	0.94011943	0.00440005	0.93394136	0.002531907	0.91656901	0.001920662
SEM-	0.01019763	8.7192E-05	0.00781961	4.0031E-05	0.00834431	3.17162E-05
SEM+		0.00026255		0.000104857		8.78867E-05
